# Polyene Carboxylic Acids from a *Streptomyces* sp. Isolated from Tibet Soil

**DOI:** 10.3390/molecules28062579

**Published:** 2023-03-12

**Authors:** Manyu Zhang, Jinwei Ren, Yuanming Luo, Weidong Xie, Erwei Li

**Affiliations:** 1 Marine College, Shandong University, Weihai 264209, China; 2 Institutional Center for Shared Technologies and Facilities, Institute of Microbiology, Chinese Academy of Sciences, Beijing 100101, China; 3 State Key Laboratory of Mycology, Institute of Microbiology, Chinese Academy of Sciences, Beijing 100101, China

**Keywords:** polyene carboxylic acid, secondary metabolites, bioactivity, NO production

## Abstract

Six new polyene carboxylic acids named serpentemycins E–J (**1**–**6**), together with three known analogs (**7**–**9**), were isolated from the fermentation medium of *Streptomyces* sp. TB060207, which was isolated from arid soil collected from Tibet, China. The structures of the new compounds were elucidated mainly on the basis of HR-ESI-MS and NMR spectroscopic analyses. The inhibitory activities of compounds **1**–**9** against NO production in LPS-activated RAW264.7 cells were evaluated. Compound **9** has an inhibition rate of 87.09% to 60.53% at concentrations ranging from 5.0 to 40.0 µM.

## 1. Introduction

Microorganism secondary metabolites have played an important role in the drug discovery of antimicrobial agents, antiviral agents, and cytotoxic and immunosuppressive compounds [1,2,3]. Actinobacteria have robust biosynthetic potential to produce bioactive compounds with broad structural diversity and commercial importance [4,5]. However, due to the frequent genetic exchange between species, the probability of the repeated discovery of known metabolites is increasing, and the difficulty of discovering new compounds is gradually increasing [6,7,8,9]. In order to increase the possibility of discovering new compounds, more and more researchers focus on actinomycetes in special environments. It is significant that new actinomycetes taxa from abnormal environments are considered very important sources of new bioactive compounds [10,11]. Actinomycetes likely have unique metabolic pathways due to their long-term special living environment (high or low temperature, acidic or alkaline medium, high salt concentration, high pressure, etc.), and have the potential to produce a variety of bioactive substances [12,13].

**Figure 1:**
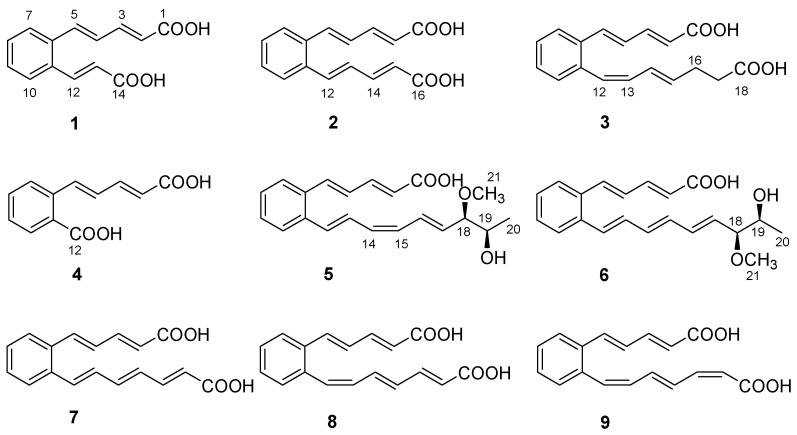
Structures of **1**−**9**.

As part of our ongoing search for new natural molecules from soil-derived actinobacteria inhabiting unique environments [14], a *Streptomyces* strain named TB060207 was collected from cold and arid soil in the low latitudes of Tibet. The actinobacteria were fermented on solid Gause’s synthetic medium (GAU medium) for 14 days and extracted by EtOAc to afford the organic extract. Subsequently, chromatographic isolation led to the identification of six new polyene carboxylic acid compounds (**1**–**6**), together with three known analogs (**7**–**9**) (Figure 1). These compounds belong to a class of rare natural products, and the ortho-substituted benzene ring is the central part of an unsaturated carboxylic acid. The analog serpentene was the first polyene carboxylic acid isolated from the filtrate of *Streptomyces* sp. Tü 3851 culture in 1993 [15]. Although discovered thirty years ago, so far only a few polyene carboxylic acid compounds (such as diacidene [16], lahorenoic acids [17], etc.) have been reported. Due to the lack of appreciable amounts of research, the biological activities of these polyunsaturated compounds have not been fully investigated. Several studies indicated this type of compound showed a broad range of activities; e.g., serpentemycin A has strong glycosyltransferase inhibitory activity [18], rubrenoic acids showed bronchodilation activity [19], and farnesylquinone possesses the effect of decreasing blood lipids [20]. In recent years, due to the development of genomic technology, the biosynthetic pathway of these compounds has been gradually explored. Their biosynthesis was proposed to be assembled via an unusual high-reduction (HR) type II polyketo synthetase (PKS) [21]. The pathway uses acetyl-CoA and malonyl-CoA as substrates, which combine with acyl carrier protein (ACP) under the action of acyltransferase (AT). Then, ketosynthetase (KS) and the chain length factor (CLF) form KS/CLF complexes that catalyze multiple rounds of decarboxylative Clarkson condensation repeatedly to form a poly-β-ketoacyl thioester attached to an ACP. After the condensation of the substrate, double bonds are formed by the catalysis of ketoreductase (KR) and dehydratase (DH), and polyene precursors are finally formed after repeated extension cycles. Isomerase (ISO) can isomerize an *E*-double bond into a *Z*-configuration, so that two carbons can be introduced within the distance required for cyclization. Subsequently, the benzene ring is formed by 6 π-electrocyclization and dehydrogenation [22,23,24].

## 2. Results and Discussion

**Figure 2:**
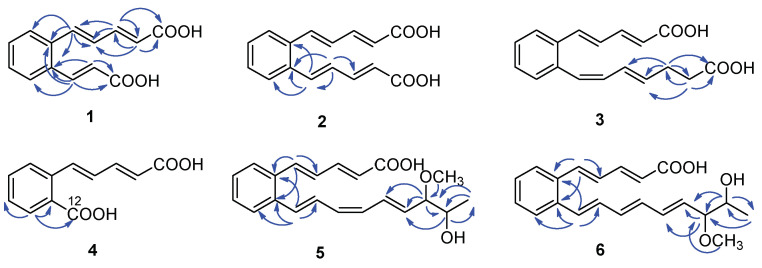
Key HMBC correlations (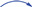) of compounds **1**–**6**.

Serpentemycin E (**1**) was obtained as a yellow amorphous powder. It has a molecular formula of C_14_H_12_O_4_ (nine degrees of unsaturation) on the basis of its HR-ESI-MS pseudomolecular ion peak at *m*/*z* 243.0657 [M−H]^−^ (calcd for C_14_H_11_O_4_ 243.0656) (Figure S4). The IR spectrum showed the absorption bands of carbonyl (1694 cm^−1^) and aromatic (1620 cm^−1^) groups. The peak at 761 cm^−1^ points to an ortho-substituted benzene. The ^1^H and ^13^C NMR spectra (Table 1) revealed the presence of two carboxy groups (*δ*_C_ 168.7, 168.6, respectively). Except for the above two carboxylic carbons, all signals belonged to aromatic and olefinic protons. These data indicated that both sides of **1** terminated with carboxy groups. The HMBC (Figure 2) correlations from H-12 to C-6, C-10, C-11, C-13, and C-14, from H-13 to C-11, C-12, and C-14 established an acrylic acid subunit that was attached to C-11. The HMBC correlations from H-2 to C-1, C-3 and C-4, from H-3 to C-1, C-2, C-4 and C-5, and from H-5 to C-4, C-6, C-7, and C-11 established a 2,4-dienoic acid subunit, and the subunit was linked to C-6. The phenyl ring was constructed of the carbon atoms C-6 to C-11, including the two quaternary carbon atoms that connected the olefinic side chain to the ring (C-6, C-11), and the aromatic methine groups C-7 (*δ*_C_ 128.0) to C-10 (*δ*_C_ 128.7). The double bond configurations of polyene chains could be determined from the ^3^*J*_H-H_ coupling constants. The larger coupling constants of all double bonds in **1** were 15.3 Hz (Table 1), which indicated that all the double bonds were *E*-configuration. Thus, the structure of **1** was established (Figure 1).

Serpentemycin F (**2**) was obtained as a yellow amorphous powder. It has a molecular formula of C_16_H_14_O_4_ (ten degrees of unsaturation) on the basis of its HR-ESI-MS pseudomolecular ion peak at *m*/*z* 269.0819 [M−H]^−^ (calcd for C_16_H_13_O_4_ 269.0814) (Figure S10). Analysis of its IR spectra, 1D NMR (Table 1), HMQC, and HMBC data revealed the presence of two carboxylic carbons (*δ*_C_ 167.6), fourteen aromatic or olefinic carbons (twelve protonated). These were very similar to **1**, so **2** and **1** have similar structures. Compared with **1**, the mass of **2** increased by 26 amu, indicating the addition of two methine carbons.

The ^13^C NMR spectrum of **2** displayed eight distinct carbon signals, which indicated that **2** had a symmetric structure. And the coupling constant value of 15.2 Hz in ^1^H NMR indicated that the configuration of all the double bonds was *E*-configured. Thus, the structure of **2** was established (Figure 1).

Serpentemycin G (**3**) has a molecular formula of C_18_H_18_O_4_ (ten degrees of unsaturation) on the basis of its HR-ESI-MS pseudomolecular ion peak at *m*/*z* 297.1129 [M−H]^−^ (calcd for C_18_H_17_O_4_ 297.1127) (Figure S15). Analysis of its IR spectra, 1D NMR (Table 1), and HMBC data revealed the presence of two carboxylic carbons (*δ*_C_ 167.6, 173.7, respectively), fourteen aromatic or olefinic carbons (twelve protonated), and two methylene carbons. Compared with the MS and NMR data with **2**, compound **3** had an increased mass of 28 amu and added two methylene carbons (*δ*_C_ 27.6, *δ*_H_ 2.26; *δ*_C_ 33.1; *δ*_H_ 2.27, respectively). In the HMBC spectrum (Figure 2), the correlations from H-16 (*δ*_H_ 2.26) to C-14, C-15, C-17, and C-18, and from H-17 (*δ*_H_ 2.27) to C-15, C-16, and C-18, showed that C-16 attached to C-15 of the olefin C-15/C-14, and C-17 attached to carboxyl C-18. According to the coupling constant (Table 1), except for 12-*Z*, the configuration of all the double bonds of **3** was *E*-configured. Thus, the structure of **3** was established (Figure 1).

Serpentemycin H (**4**) was obtained as a yellow amorphous powder. It has a molecular formula of C_12_H_10_O_4_ (eight degrees of unsaturation) on the basis of its HR-ESI-MS pseudomolecular ion peak at *m*/*z* 217.0501 [M−H]^−^ (calcd for C_12_H_9_O_4_ 217.0501) (Figure S20). Compounds **4** differ from **1** by 26 amu, indicating the loss of two methine carbons. This is in agreement with IR spectra, 1D NMR (Table 2), and HMBC data. The carboxyl C-12 (*δ*_C_ 167.5) was attached to C-11, as confirmed by the HMBC correlations from H-10 (*δ*_H_ 7.83) to C-9, C-11, and C-12. According to the coupling constant, the configuration of all the double bonds of **4** was *E*-configured. Thus, the structure of **4** was established as shown (Figure 1).

Serpentemycin I (**5**) was obtained as a yellow amorphous powder. It has a molecular formula of C_21_H_24_O_4_ (ten degrees of unsaturation) on the basis of its HR-ESI-MS pseudomolecular ion peak at *m*/*z* 339.1598 [M−H]^−^ (calcd for C_21_H_23_O_4_ 339.1596) (Figure S26). The ^1^H and ^13^C NMR spectra (Table 2) revealed the presence of two methyl groups (including one methoxy), two sp^3^ methylene groups, sixteen aromatic or olefinic carbons (fourteen protonated), and one carboxylic carbon. The ^1^H-^1^H COSY spectrum reveals three independent spin systems: a pentadienoic acid moiety, a disubstituted benzene ring, and an 8-hydroxy-7-methoxy-1,3,5-nonatrienyl residue or a 7-hydroxy-8-methoxy-1,3,5-nonatrienyl residue. The positions of hydroxyl and methoxy groups cannot be determined, except that they are connected to methylene. In the HMBC spectrum, H-21 is only associated with C-18, while the correlation of H-18 with C-16, C-17, C-19, C- 20, and C-21 indicates that this subunit is an 8-hydroxy-7-methoxy-1,3,5-nonatrienyl residue. The HMBC correlations (Figure 2) from H-5 to C-4, C-6, and C-11 indicated that C-5 was directly attached to the quaternary carbon atom C-6 of the benzene ring. The HMBC correlations from H-12 to C-6, C-10, C-11, and C-13 indicated that C-12 was directly attached to the second quaternary carbon atom C-11 on the ring. According to the coupling constant (Table 2), the configuration of all double bonds of **5** except 14-*Z* was *E*-type. Thus, the planar structure of **5** was established (Figure 1).

**Figure 3:**
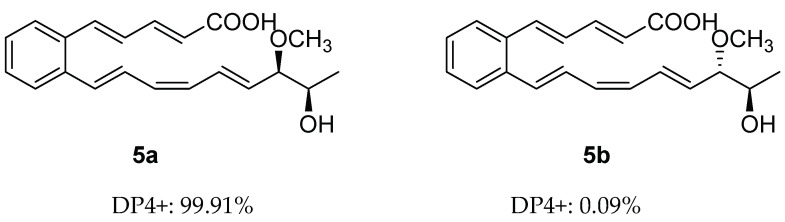
DP4+ probability of two plausible epimers **5a** and **5b** of **5** (**5a**: rel-(18*S*,19*S*), **5b:** rel-(18*S*,19*R*)).

Despite the presence of two stereogenic carbons (C-18 and C-19) in its molecule, the optical rotation value of **5** is close to zero, which indicates that **5** is a racemic mixture. Calculated NMR with DP4+ analysis was used to establish the relative configuration of **5**. There were two possible isomers of **5**, respectively, **5a** (rel-(18*S*,19*S*)) and **5b** (rel-(18*S*,19*R*)) (Figure 3). Chemical shifts of isomers **5a** and **5b** were predicted using the GIAO method, with DFT calculations in DMSO, using the PCM model at the B3LYP/6-31+G(d,p) level (Tables S1, S5 and S6). Then, the calculated chemical shifts of these two possible isomers (**5a** and **5b**) were compared with the experimental data of **5**, and statistical analysis was carried out using the DP4+ method (Table S3). The analysis of DP4+ showed that isomer **5a** was the most reasonable relative configuration, and the combined probability of NMR data was 99.91% (Figures S34 and S36). Thus, the relative configuration of **5** was determined as **5a** (Figure 3).

Serpentemycin J (**6**) was obtained as a yellow amorphous powder. The molecular formula was determined as C_21_H_24_O_4_ (ten degrees of unsaturation) by HR-ESI-MS at *m*/*z* 339.1600 [M−H]^−^ (Figure S32), which was the same as that of **5**. The ^1^H and ^13^C NMR data (Table 2) and 2D NMR were very similar, indicating that **6** was an isomer of **5**. According to the coupling constant (Table 2), the configurations of all double bonds of **6** were found to be *E*-type. Thus, the planar structure of **6** was established as shown in Figure 1.

**Figure 4:**
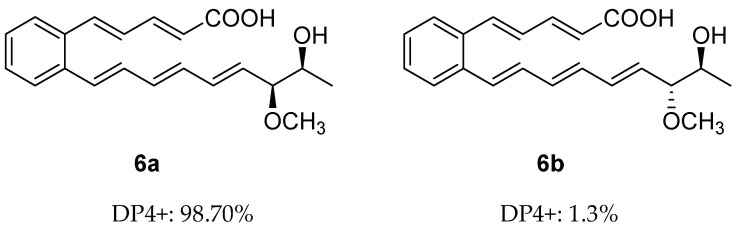
DP4+ probability of two plausible epimers **6a** and **6b** of **6** (**6a**: rel-(18*R*,19*R*). **6b:** rel-(18*S*,19*R*)).

Similar to **5**, serpentemycin J (**6**) has two stereogenic carbons (C-18, C-19) and its optical rotation value is close to zero, indicating that **6** is racemic as well. The relative configuration of C-18/C-19 of **6** was also determined by GIAO NMR chemical shift calculation, and then DP4+ analysis was performed (Tables S2, S4, S7 and S8). The DP4+ analysis results showed that the relative configuration of **6** was **6a** (rel-(18*R*,19*R*)) instead of **6b** (rel-(18*S*,19*R*)) (Figures S35 and S37). Thus, the relative configuration of **6** was determined as **6a** (Figure 4).

The structures of compounds **7**–**9** (Figure 1) were determined by comparison of their spectroscopic data with those in the literature [15,25], which reported that polyene carboxylic acids are sensitive to light and heat due to the configuration isomerization or cyclization of double bonds.

**Figure 5:**
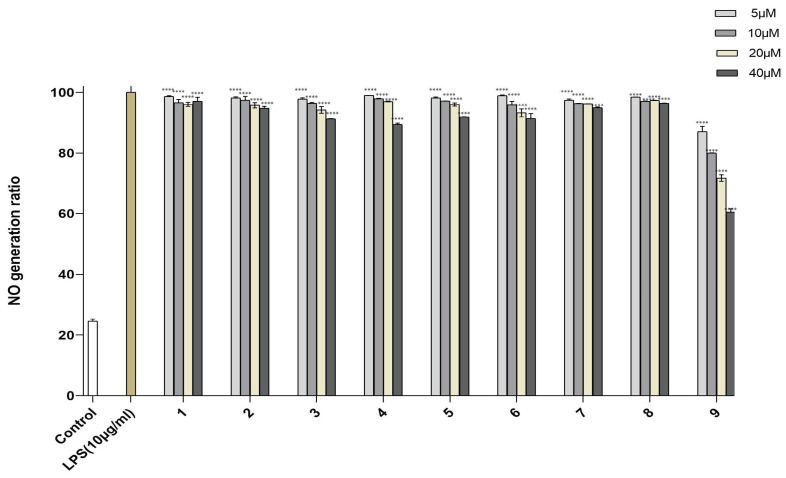
NO production in RAW246.7 cells treated with compounds **1**–**9**. Control was used for blank control, LPS-induced NO production was used for positive control and represented at 100%. Data are expressed as mean ± SD from triplicate experiments. **** *p* < 0.0001 accepted as significant.

Human monocytes and macrophages are exquisitely sensitive to the endotoxin lipopolysaccharide (LPS) and induce inflammatory reactions by expressing many inflammatory cytokines, such as TNF-α, IL-1b, and NO. Therefore, inhibiting inflammatory cytokines is important for curtailing inflammatory disorders [26,27,28]. Compounds **1**–**9** were evaluated for their inhibitory activity of NO production in LPS-activated RAW264.7 cells. To avoid the possible effect of reduced viability on the NO production induced by cytotoxic activity, the cytotoxic activities of **1**–**9** on RAW264.7 cells were tested by MTT assay (Table S9). These compounds showed no obvious cytotoxicity at the tested concentrations [29]. The results showed that compounds **1**–**8** had little inhibitory activity against NO production in LPS-activated RAW264.7 cells. Compound **9** had the largest potency of inhibiting NO production, with an inhibition rate of 60.53% at 40.0 µM (Figure 5).

The cytotoxic activity of compounds **1**–**9** was evaluated by MTT assay. These compounds showed no obvious cytotoxicity at concentrations of 20 μM against the breast cancer MCF-7 cells and had no cytotoxicity on the RAW264.7 cells at concentrations of 200 μM (Table S9).

## 3. Materials and Methods

### 3.1. General Experimental Procedures

Infrared spectra were recorded with a Nicolet IS5 FT-IR spectrophotometer (Waltham, MA, USA). The UV data were obtained on a Thermo Scientific Genesys 10S spectrophotometer (Madison, WI, USA). Optical rotations were measured with an Anton Paar MCP 200 Automatic Polarimeter (Graz, Austria). The NMR data were performed on a Bruker Avance-500 MHz spectrometer (Bruker, Rheinstetten, Germany). HRESIMS data were performed on a Waters ACQUITY UPLC I-Class Plus-Xevo G2-XS QToF (Manchester, UK). Semipreparative HPLC isolation was conducted with an Agilent 1200 HPLC system using an ODS column (YMC-Triart C18, 10 mm × 250 mm, YMC Co., Ltd., Tokyo, Japan) with a flow rate of 2.0 mL/min. Silica gel (300–400 mesh), used in column chromatography (CC), and silica gel GF 254 (10–40 µm), used in thin layer chromatography (TLC), were purchased from Qingdao Marine Chemical Factory, Qingdao, China.

### 3.2. Strain and Fermentation

Actinomycetes (TB060207) were isolated from the arid soil collected in low-latitude areas of Tibet, China. The strain was first placed on Petri dishes containing 60 L Gause’s synthetic medium (20 mL/dish) (GAU; 0.01 g/L FeSO_4_·H_2_O, 0.5 g/L NaCl, 0.5 g/L K_2_HPO_4_·H_2_O, 0.5 g/L MgSO_4_·H_2_O, 20g/L amylogen, and 1 g/L KNO_3_) containing nalidixic acid (20 μg/mL) and potassium dichromate (100 μg/mL). The plates were cultured for 14 days at 37 °C.

### 3.3. Extraction and Isolation

At the end of the fermentation, the culture was extracted with EtOAc (each 60 L, four times) and vacuum-dried to afford the crude extract (15.0 g). The extract was fractionated by silica gel column chromatography (150 g, 200–300 mesh), eluted with CH_2_Cl_2_-MeOH (100:1, 50:1, 20:1, 10:1, and methanol) to give six fractions (Frs.1–Frs.6). Fraction Frs.4 (2.62 g) was loaded on ODS CC, eluted with 10–100% MeOH to afford twelve fractions (Frs.4.1–Frs.4.12). The subfraction Frs.4.2 (61.1 mg) was subjected to Sephadex LH-20 CC, eluted with MeOH to generate three fractions (Frs.4.2.1, Frs.4.2.3). The Frs.4.2.1 (32.0 mg) was further purified by RP-HPLC (70% MeOH in H_2_O with 0.1% HCOOH for 30 min; 2.0 mL/min) to afford **4** (3.8 mg, *t*_R_ 27.3 min). The Frs.4.3 was purified by RP HPLC (72% MeOH in H_2_O with 0.1% HCOOH for 30 min; 2.0 mL/min) to afford **7** (3.2 mg, *t*_R_ 29.3 min). The subfraction Frs.4.4 (148.6 mg) was loaded on Sephadex LH-20 CC, eluted with MeOH to afford two tertiary fractions (Frs.4.4.1, Frs.4.4.2). The Frs.4.4.2 (42.0 mg) was purified by RP HPLC (64% MeOH in H_2_O with 0.1% HCOOH for 30 min; 2.0 mL/min) to afford **1** (3.8 mg, *t*_R_ 23.7min). The subfraction Frs.4.6 (66.3 mg) was purified by RP HPLC (74% MeOH in H_2_O with 0.1% HCOOH for 35 min; 2.0 mL/min) to afford **8** (4.2 mg, *t*_R_ 28.2 min) and **3** (3.1 mg, *t*_R_ 30.6 min). The subfraction Frs.4.8 (88.3 mg) was subjected to Sephadex LH-20 CC, eluted with MeOH to generate two fractions (Frs.4.8.1, Frs.4.8.2). The Frs.4.8.1 (42.3 mg) was further purified by RP-HPLC (78% MeOH in H_2_O with 0.1% HCOOH for 30 min; 2.0 mL/min) to give **2** (3.0 mg, *t*_R_ 27.8 min) and **9** (3.0 mg, *t*_R_ 29.6 min). The subfraction Frs.4.10 (215.8 mg) was loaded on Sephadex LH-20 CC and eluted with MeOH to generate three fractions (Frs.4.10.1, Frs.4.10.3). Part of fraction Frs.4.10.1 (33.1 mg) was further purified by RP HPLC (76% MeOH in H_2_O with 0.1% HCOOH for 30 min; 2.0 mL/min) to give **5** (3.4 mg, *t*_R_ 32.4 min) and **6** (3.0 mg, *t*_R_ 32.6 min). These compounds are unstable to light and heat due to their open-chain polyenes with multiple conjugated double bonds. Thus, the isolations were performed almost completely in the dark.

#### 3.3.1. Serpentemycin E (**1**)

Yellow amorphous powder; UV (MeOH) *λ*_max_ (log*ε*) 272 (3.07), 317 (3.01) nm. IR (neat) *ν*_max_ 3027, 1694, 1620, 761 cm^−1^. ^1^H NMR (500 MHz, DMSO-*d*_6_) and ^13^C NMR (125 MHz, DMSO-*d*_6_) data, Table 1; HR-ESI-MS *m*/*z* 243.0657 [M−H]^−^ (calcd for C_14_H_11_O_4_, 243.0656).

#### 3.3.2. Serpentemycin F (**2**)

Yellow amorphous powder; UV (MeOH) *λ*_max_ (logε) 291 (2.898), 332 (2.618) nm. IR (neat) *ν*_max_ 3045, 1682, 1617, 1280, 747 cm^−1^. ^1^H NMR (500 MHz, DMSO-*d*_6_) and ^13^C NMR (125 MHz, DMSO-*d*_6_) data, Table 1; HR-ESI-MS *m*/*z* 269.0819 [M−H]^−^ (calcd for C_16_H_13_O_4_, 269.0819).

#### 3.3.3. Serpentemycin G (**3**)

Yellow amorphous powder; UV (MeOH) *λ*_max_ (logε) 318 (2.106) nm. IR (neat) *ν*_max_ 3029, 1699, 1619, 1276, 758 cm^−1^. ^1^H NMR (500 MHz, DMSO-*d*_6_) and ^13^C NMR (125 MHz, DMSO-*d*_6_) data, Table 1; HR-ESI-MS *m*/*z* 297.1129 [M−H]^−^ (calcd for C_18_H_17_O_4_, 297.1127).

#### 3.3.4. Serpentemycin H (**4**)

Yellow amorphous powder; UV (MeOH) *λ*_max_ (logε) 307 (2.648) nm. IR (neat) *ν*_max_ 2947, 1682, 1618, 1312, 1271, 750 cm^−1^. ^1^H NMR (500 MHz, DMSO-*d*_6_) and ^13^C NMR (125 MHz, DMSO-*d*_6_) data, Table 2; HR-ESI-MS *m*/*z* 217.0501 [M−H]^−^ (calcd for C_12_H_9_O_4_, 217.0501).

#### 3.3.5. Serpentemycin I (**5**)

Yellow amorphous powder; [*α*]_D_^25^ ± 0 (c 0.1, MeOH); UV (MeOH) *λ*_max_ (logε) 298 (1.654), 331 (1.355) nm. IR (neat) *ν*_max_ 2931, 1698, 1622, 1251, 999, 756 cm^−1^. ^1^H NMR (500 MHz, DMSO-*d*_6_) and ^13^C NMR (125 MHz, DMSO-*d*_6_) data, Table 2; HR-ESI-MS *m*/*z* 339.1598 [M−H]^−^ (calcd for C_21_H_23_O_4_, 339.1596).

#### 3.3.6. Serpentemycin J (**6**)

Yellow amorphous powder; [*α*]_D_^25^ ± 0 (c 0.1, MeOH); UV (MeOH) *λ*_max_ (logε) 294 (0.901), 330 (0.764) nm. IR (neat) *ν*_max_ 2931, 1697, 1622, 1251, 999, 756 cm^−1^. ^1^H NMR (500 MHz, DMSO-*d*_6_) and ^13^C NMR (125 MHz, DMSO-*d*_6_) data, Table 2; HR-ESI-MS *m*/*z* 339.1600 [M−H]^−^ (calcd for C_21_H_23_O_4_, 339.1596).

### 3.4. Computational NMR Chemical Shift Calculations for DP4 Analysis

All theoretical calculations were performed using the Gaussian 16 program package [30]. A conformation search of all possible isomers in MMFF 94 molecular force fields was carried out by GMMX. The conformational isomers were optimized using the DFT method at the B3LYP/6-31+G (d,p) level in gas. Then, according to the frequency and Boltzmann distribution theory, further conformation analyses were carried out to remove the irrational and unstable conformers, and at the same time, room-temperature equilibrium proportions were calculated according to the Boltzmann distribution law. The gauge-independent atomic orbital (GIAO) calculations of NMR shielding constants for all stable conformers using the density functional theory (DFT) method in DMSO, using the polarizable continuum model (PCM) model at the B3LYP/6-31G(d) level. The obtained shielding constants (including ^13^C and ^1^H) were directly statistically analyzed with experimental chemical shifts, and DP4+ probability is used. Using DP4+ probability, the obtained shielding constants (including ^13^C and ^1^H) were directly statistically analyzed by experimental chemical shift [31]. Finally, DP4+ probability analysis is carried out.

### 3.5. MTT Assay

The cytotoxic activity of compounds **1**–**9** against breast cancer cells MCF-7 and mouse mononuclear macrophage RAW264.7 was determined using the 3-(4,5-dimethylthiazole-2-yl)-2,5-diphenyltetrazolium bromide (MTT) assay. Doxorubicin was used as a positive control drug, and deionized H_2_O with the same DMSO concentration was used as a parallel control [32,33].

### 3.6. NO Determination Assay

RAW264.7 cells were cultured in 96-well plates (5000 cells per well) with 100 µL complete culture media in 5% CO_2_ at 37 °C, after overnight culturing, cells were added the test compounds (3.75, 7.5, 15, and 30 μM) for 30 min. The cells were treated with LPS (2 µg/mL) for 24 h. The supernatant was mixed with an equal volume of Griess reagent, and after incubating the mixed solution at room temperature for 5 min. Absorbance at 570 nm was then determined using an enzyme labeling reagent [26,34].

## 4. Conclusions

Nine polyene carboxylic acids were isolated from *Streptomyces* sp. TB060207, including six new ones, serpentemycin E–J (**1**–**6**), and three known analogs (**7**–**9**). Among them, compound **9** showed inhibitory activities against NO production in LPS-activated RAW264.7 cells with an inhibition rate of 60.53% at 40.0 µM. These compounds are open-chain polyenes with more than two conjugated double bonds centered on ortho-substituted benzene rings and belong to serpentene derivatives [15]. Because their structures are very unique and few compounds have been found at present, biosynthetic pathways have not been fully understood. The discovery of these new compounds is of great significance for further study of their biosynthesis and activity.

## Figures and Tables

**Table 1:** NMR data for **1**−**3.**

Position	1a		2		3	
	*δ*_C_ ^a^	*δ*_H_ ^b^ (*J* in Hz)	*δ*_C_ ^a^	*δ*_H_ ^b^ (*J* in Hz)	*δ*_C_ ^a^	*δ*_H_ ^b^ (*J* in Hz)
1	168.7 C		167.6 C		167.6 C	
2	124.4 CH	6.05 (d, 15.3)	122.9 CH	6.04 (d, 15.2)	122.7 CH	6.02 (d, 15.5)
3	145.5 CH	7.49 (dd, 15.3, 11.0)	144.4 CH	7.45 (dd, 15.2, 11.0)	144.2 CH	7.34 (m)
4	131.2 CH	7.02 (dd, 15.3, 11.0)	128.9 CH	7.04 (dd, 15.2, 11.0)	127.8 CH	7.03 (dd, 15.5, 11.2)
5	137.4 CH	7.47 (d, 15.3)	136.3 CH	7.57 (d, 15.2)	137.2 CH	7.14 (d, 15.5)
6	136.9 C		134.5 C		134.2 C	
7	128.0 CH	7.69 (m)	126.4 CH	7.71 (m)	125.8 CH	7.71 (m)
8	131.4 CH	7.43 (m)	129.0 CH	7.35 (m)	127.5 CH	7.31 (m)
9	130.3 CH	7.37 (m)			128.5 CH	7.32 (m)
10	128.7 CH	7.74 (m)			130.1 CH	7.23 (m)
11	134.1 C				136.4 C	
12	141.8 CH	7.95 (d, 15.3)			126.4 CH	6.53 (d, 11.2)
13	123.2 CH	6.43 (d, 15.3)			131.1 CH	6.35 (dd, 11.2, 11.2)
14	168.6 C				136.0 CH	6.22 (dd, 15.5, 11.2)
15					126.1 CH	5.92 (m)
16					27.6 CH2	2.26 (m)
17					33.1 CH2	2.27 (m)
18					173.7 C	

^a^ Recorded at 125 MHz in DMSO-*d*_6_. ^b^ Recorded at 500 MHz in DMSO-*d*_6._

**Table 2:** NMR data for **4**–**6.**

Position	4		5		6	
	*δ*_C_ ^a^	*δ*_H_ ^b^ (*J* in Hz)	*δ*_C_ ^a^	*δ*_H_ ^b^ (*J* in Hz)	*δ*_C_ ^a^	*δ*_H_ ^b^ (*J* in Hz)
1	168.4 C		167.6 C		167.6 C	
2	123.0 CH	6.05 (d, 15.2)	122.4 CH	6.03 (d, 15.3)	122.4 CH	6.03 (d, 15.3)
3	144.3 CH	7.32 (dd, 15.2, 11.0)	144.6 CH	7.45 (dd, 15.3, 11.0)	144.6 CH	7.45 (dd, 15.3, 11.0)
4	128.6 CH	7.02 (dd, 15.6, 11.0)	128.4 CH	7.00 (dd, 15.3, 11.0)	128.4 CH	7.00 (dd, 15.3, 11.0)
5	138.2 CH	7.69 (d, 15.6)	136.9 CH	7.49 (d, 15.3)	136.9 CH	7.49 (d, 15.3)
6	136.4 C		133.7 C		133.7 C	
7	126.9 CH	7.77 (m)	126.3 CH	7.29 (m)	126.3 CH	7.29 (m)
8	130.6 CH	7.56 (m)	127.7 CH	7.63 (m)	127.7 CH	7.63 (m)
9	131.8 CH	7.42 (m)	128.9 CH	7.63 (m)	129.0 CH	7.63 (m)
10	130.3 CH	7.83 (m)	125.8 CH	7.29 (m)	125.8 CH	7.29 (m)
11	128.6 C		135.7 C		135.7 C	
12	167.5 C		128.9 CH	7.09 (d, 15.3)	129.0 CH	7.09 (d, 15.3)
13			131.6 CH	6.89 (dd, 15.3, 9.7)	131.6 CH	6.89 (dd, 15.3, 9.7)
14			133.4 CH	6.53 (dd, 10.1, 9.7)	133.4 CH	6.53 (dd, 15.3, 9.7)
15			133.3 CH	6.48 (dd, 10.1, 9.7)	133.3 CH	6.48 (dd, 15.3, 9.7)
16			133.2 CH	6.34 (dd, 15.3, 9.7)	133.3 CH	6.34 (dd, 15.3, 9.7)
17			132.6 CH	5.67 (dd, 15.3, 7.6)	132.0 CH	5.67 (dd, 15.3, 7.7)
18			86.2 CH	3.48 (m)	86.1 CH	3.49 (m)
19			68.3 CH	3.62 (m)	68.0 CH	3.63 (m)
20			19.3 CH3	1.02 (d, 6.4)	18.7 CH3	0.97 (d, 6.4)
21			56.1 MeO	3.20 (s)	56.2 MeO	3.20 (s)

^a^ Recorded at 125 MHz in DMSO-*d*_6_. ^b^ Recorded at 500 MHz in DMSO-*d*_6._

## Data Availability

The data presented in this study are available on request from the corresponding authors.

## Supplementary Materials

The following supporting information can be downloaded at: https://www.mdpi.com/article/10.3390/molecules28062579/s1, This section includes 1D, 2D NMR spectra, IR spectrum and the HR-ESI-MS for **1**–**6**; computational data of compounds **5**−**6**. Figures S1–S33: IR, HR-ESI-MS, 1D and 2D NMR of compounds **1**–**6**. Figure S34–S35: Experimental chemical shifts and calculated shielding tensors for PD4+ probability analysis for compound **5** and **6**. Figure S36–S37: DP4+ probability analysis of **5** and **6**. Table S1: Experimental (Exp.) and calculated (Cal.) 1H and 13C chemical shift values of **5** and its possible isomers **5a** and **5b** used for DP4+ analysis. Table S2: Experimental (Exp.) and calculated (Cal.) 1H and 13C chemical shift values of 6 and its possible isomers **6a** and **6b** used for DP4+ analysis. Table S3–S4: DFT-optimized structures and thermodynamic parameters for low-energy conformers of **5a**, **5b**, **6a** and **6b**. Table S5–S8. Optimized Z-matrixes of **5a**, **5b**, **6a** and **6b** in the gas phase (Å) at B3LYP/6-31G(d) level. Table S9. Cytotoxicity of **1**–**9** against RAW264.7 and MCF-7 cells (IC50 μM).

## Sample Availability

Samples of the compounds are available from the authors.
