# Supplementary material for: Polyene Carboxylic Acids from a Streptomyces sp. Isolated from Tibet Soil
Source: Molecules. 2023 Mar 12;28(6):2579. doi: 10.3390/molecules28062579 (PMC10054270; doi:10.3390/molecules28062579)
Supplement: Supplementary file 1 [file molecules-28-02579-s001.zip › molecules-2248344-supplementary.pdf]

Supplementary Materials

Polyene Carboxylic Acids from a *Streptomyces* sp. Isolated from Tibet Soil

Manyu Zhang [1,2], Jinwei Ren [3], Yuanming Luo [2], Weidong Xie [1,*] and Erwei Li [2,*]

[1] Marine College, Shandong University, Weihai 264209, China; 18737809192@163.com (M-Y.Z.)
[2] Institutional Center for Shared Technologies and Facilities, Institute of Microbiology, Chinese Academy of Sciences, Beijing 100101, China; luoym@im.ac.cn (Y-M.L.)
[3] State Key Laboratory of Mycology, Institute of Microbiology, Chinese Academy of Sciences, Beijing 100101, China; renjw@im.ac.cn (J-W.R.)
* Correspondence: wdxie@sdu.edu.cn (W-D.X.); liew@im.ac.cn (E-W.L.); Tel.: +86-631-568-8303 (W-D.X.); +86-10-6480-6141 (E-W.L.)

Keywords: polyene carboxylic acid/secondary metabolites/bioactivity/NO production

Contents

Figure S1. ^{1}H-NMR spectrum (500 MHz, DMSO-d_6) of **1**.
Figure S2. ^{13}C-NMR spectrum (125 MHz, DMSO-d_6) of **1**.
Figure S3. HMBC spectrum (500 MHz, DMSO-d_6) of **1**.
Figure S4. HR-ESI-MS of **1**.
Figure S5. IR spectrum of **1.**
Figure S6 ^{1}H-NMR spectrum (500 MHz, DMSO-d_6) of **2**.
Figure S7. ^{13}C-NMR spectrum (125 MHz, DMSO-d_6) of **2**.
Figure S8. HMQC spectrum (500 MHz, DMSO-d_6) of **2**.
Figure S9. HMBC spectrum (500 MHz, DMSO-d_6) of **2**.
Figure S10. HR-ESI-MS of **2**.
Figure S11. IR spectrum of **2.**
Figure S12. ^{1}H-NMR spectrum (500 MHz, DMSO-d_6) of **3**.
Figure S13. ^{13}C-NMR spectrum (125 MHz, DMSO-d_6) of **3**.
Figure S14. HMBC spectrum (500 MHz, DMSO-d_6) of **3**.
Figure S15. HR-ESI-MS of **3**.
Figure S16. IR spectrum of **3.**
Figure S17. ^{1}H-NMR spectrum (500 MHz, DMSO-d_6) of **4**.
Figure S18. ^{13}C-NMR spectrum (125 MHz, DMSO-d_6) of **4**
Figure S19. HMBC spectrum (500 MHz, DMSO-d_6) of **4**.
Figure S20. HR-ESI-MS of **4**.
Figure S21. IR spectrum of **4.**
Figure S22. ^{1}H-NMR spectrum (500 MHz, DMSO-d_6) of **5**.
Figure S23. ^{13}C-NMR spectrum (125 MHz, DMSO-d_6) of **5**.
Figure S24. ^{1}H-^{1}H COSY spectrum (500 MHz, DMSO-d_6) of **5**.
Figure S25. HMBC spectrum (500 MHz, DMSO-d_6) of **5**.
Figure S26. HR-ESI-MS of **5**.
Figure S27. IR spectrum of **5.**
Figure S28. ^{1}H-NMR spectrum (500 MHz, DMSO-d_6) of **6**.
Figure S29. ^{13}C-NMR spectrum (125 MHz, DMSO-d_6) of **6**.
Figure S30. ^{1}H-^{1}H COSY spectrum (500 MHz, DMSO-d_6) of **6**.
Figure S31. HMBC spectrum (500 MHz, DMSO-d_6) of **6**.
Figure S32. HR-ESI-MS of **6**.
Figure S33. IR spectrum of **6.**
Figure S34. Experimental chemical shifts and calculated shielding tensors for PD4+ probability analysis for compound **5**. (isomer **5a**: rel-(18*S*,19*S*)-**5**; isomer **5b**: rel-(18*S*,19*R*)-**5**)
Figure S35. Experimental chemical shifts and calculated shielding tensors for PD4+ probability analysis for compound **6** (isomer **6a**: rel-(18*R*,19*R*)-**6**; isomer **6b**: rel-(18*S*,19*R*)-**6**).
Figure S36. DP4+ probability analysis of **5** with isomers **5a** and **5b**.
Figure S37. DP4+ probability analysis of **6** with isomers **6a** and **6b**.
Table S1. Experimental (Exp.) and calculated (Cal.) ^{1}H and ^{13}C chemical shift values of **5** and its possible isomers **5a** and **5b** used for DP4+ analysis.
Table S2. Experimental (Exp.) and calculated (Cal.) ^{1}H and ^{13}C chemical shift values of **6** and its possible isomers **6a** and **6b** used for DP4+ analysis.
Table S3. DFT-optimized structures and thermodynamic parameters for low-energy

conformers of **5a** and **5b**.
Table S4. DFT-optimized structures and thermodynamic parameters for low-energy conformers of **6a** and **6b**.
Table S5. Optimized Z-matrixes of **5a** in the gas phase (Å) at B3LYP/6-31G(d) level.
Table S6. Optimized Z-matrixes of **5b** in the gas phase (Å) at B3LYP/6-31G(d) level.
Table S7. Optimized Z-matrixes of **6a** in the gas phase (Å) at B3LYP/6-31G(d) level.
Table S8. Optimized Z-matrixes of **6b** in the gas phase (Å) at B3LYP/6-31G(d) level.
Table S9. Cytotoxicity of **1–9** against RAW264.7 and MCF-7 cells (IC_{50} μM).

Figure S1. ¹H-NMR spectrum (500 MHz, DMSO-d_6) of **1**.

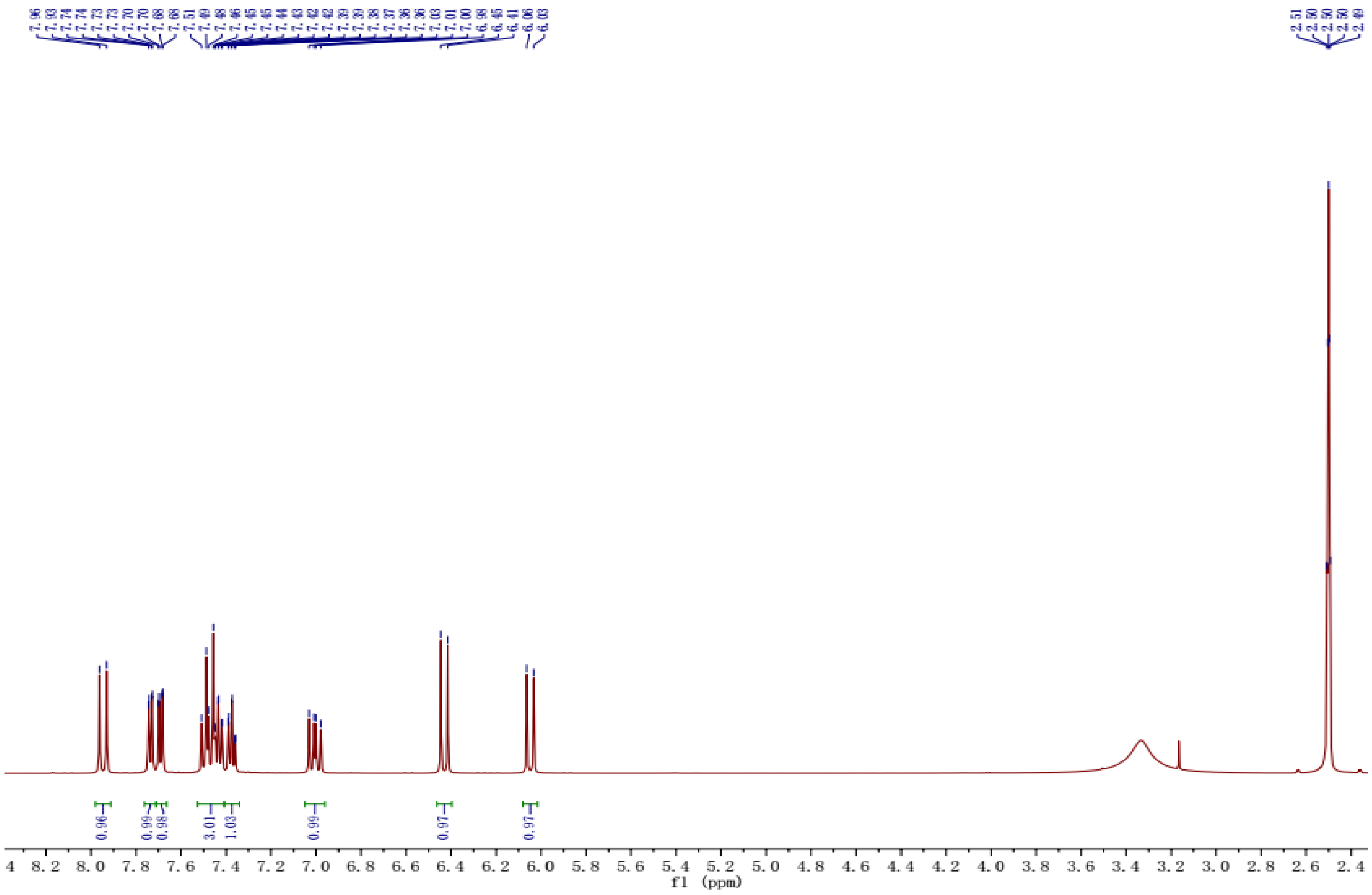

Figure S2. ^{13}C-NMR spectrum (125 MHz, DMSO-d_6) of **1**.

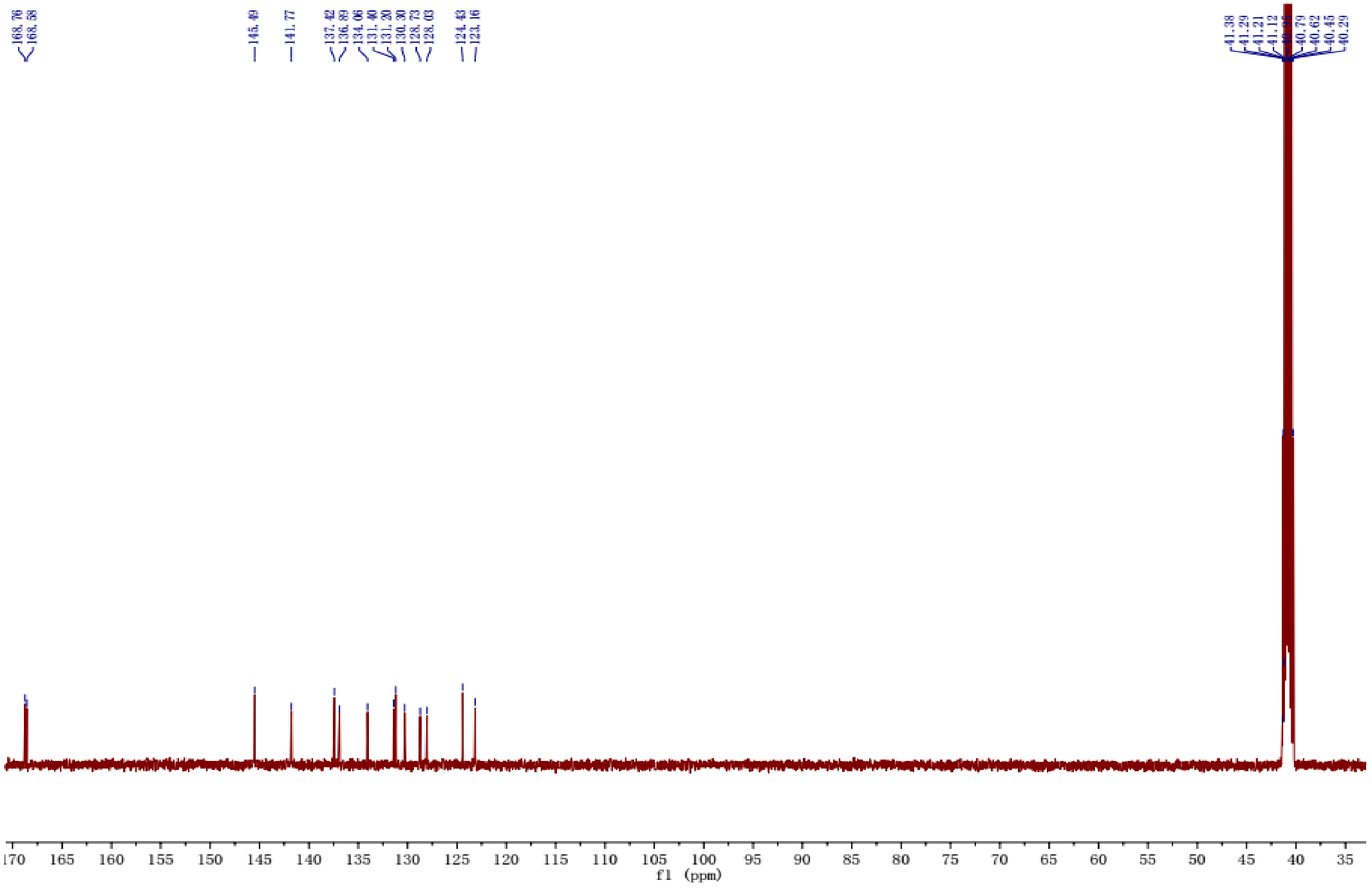

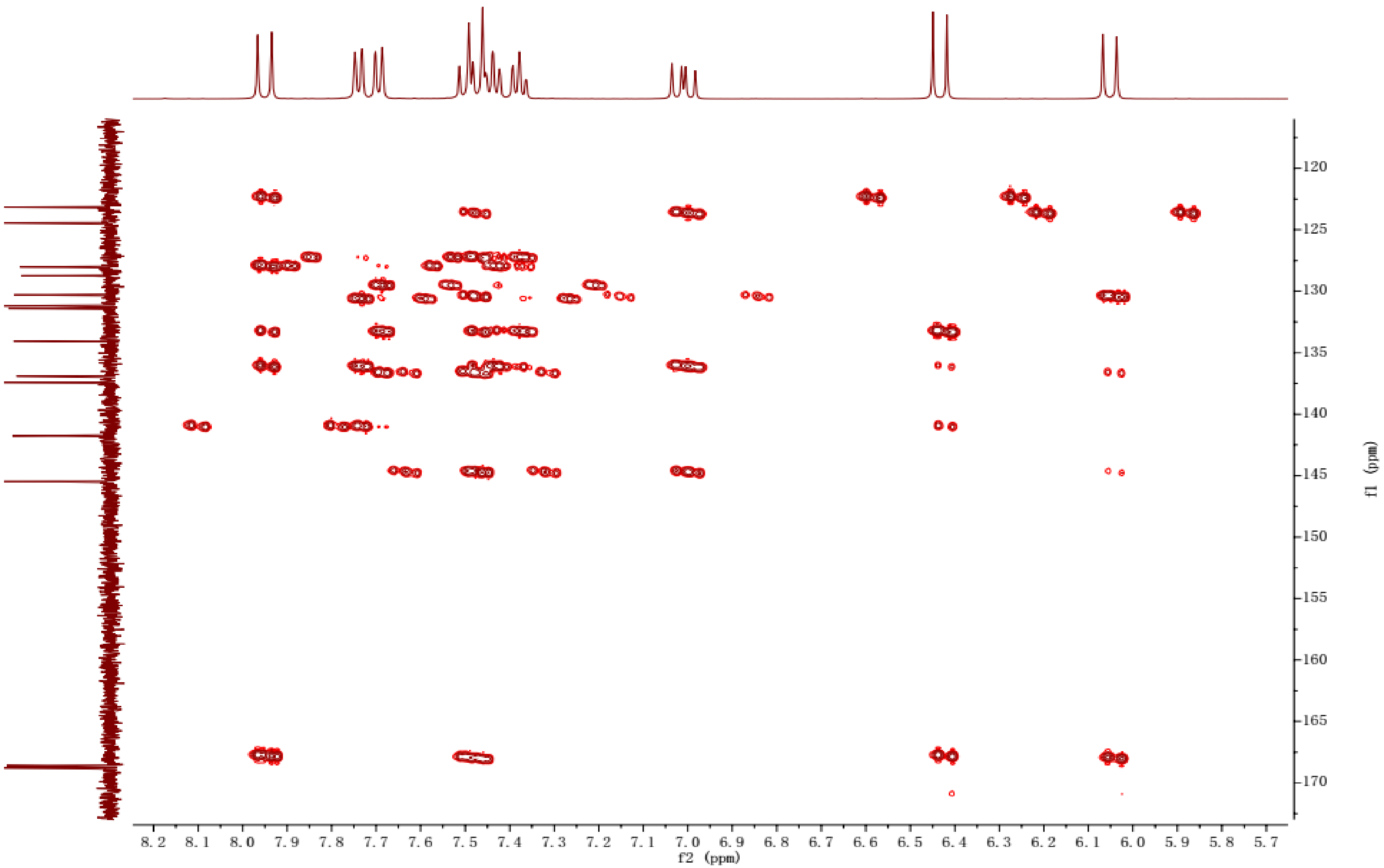

Figure S3. HMBC spectrum (500 MHz, DMSO-d_6) of **1**.

Figure S4. HR-ESI-MS of 1.

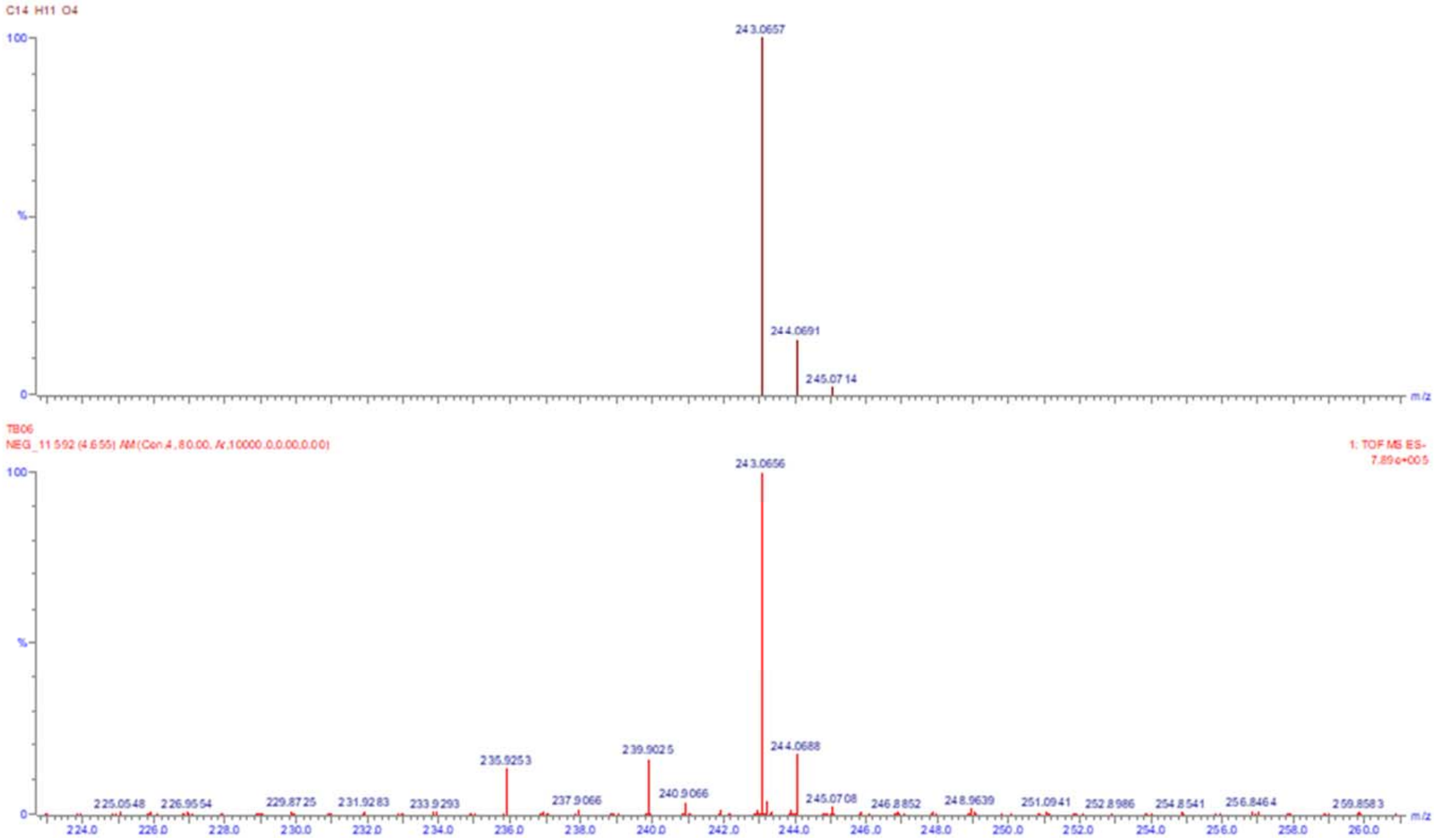

Figure S5. IR spectrum of **1.**

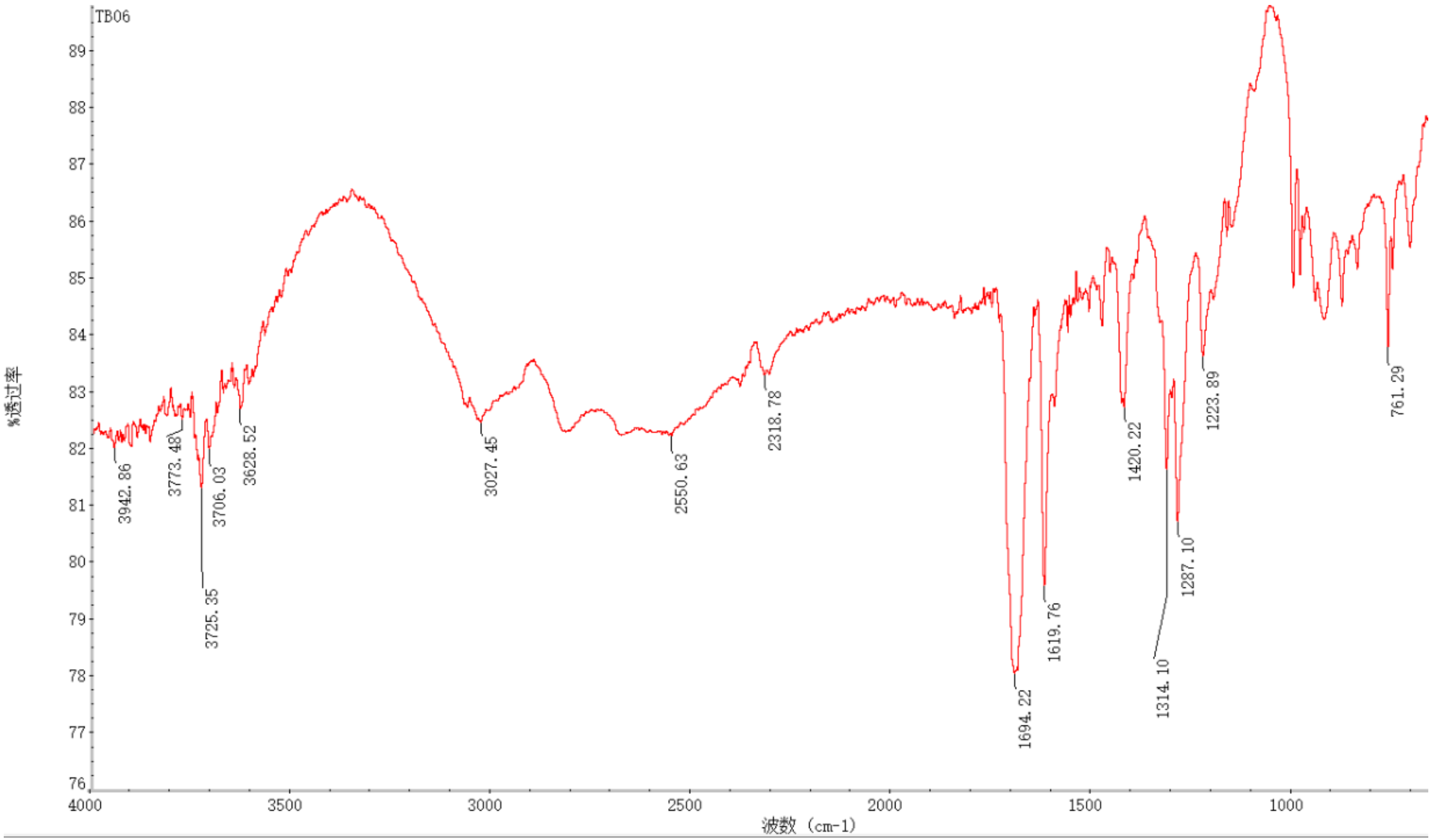

Figure S6 ^{1}H-NMR spectrum (500 MHz, DMSO-d_6) of **2**.

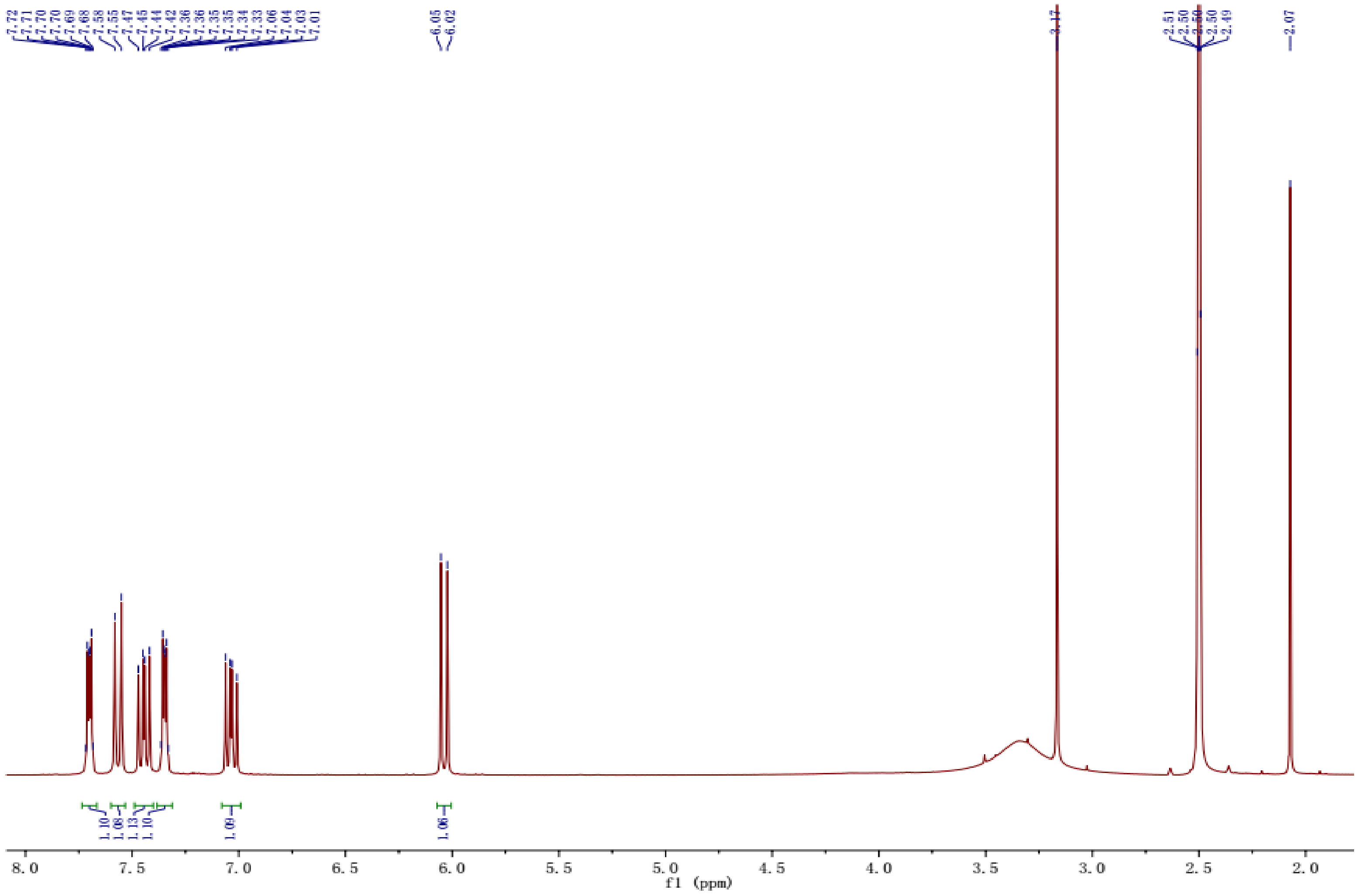

Figure S7. ^{13}C-NMR spectrum (125 MHz, DMSO-d_6) of **2**.

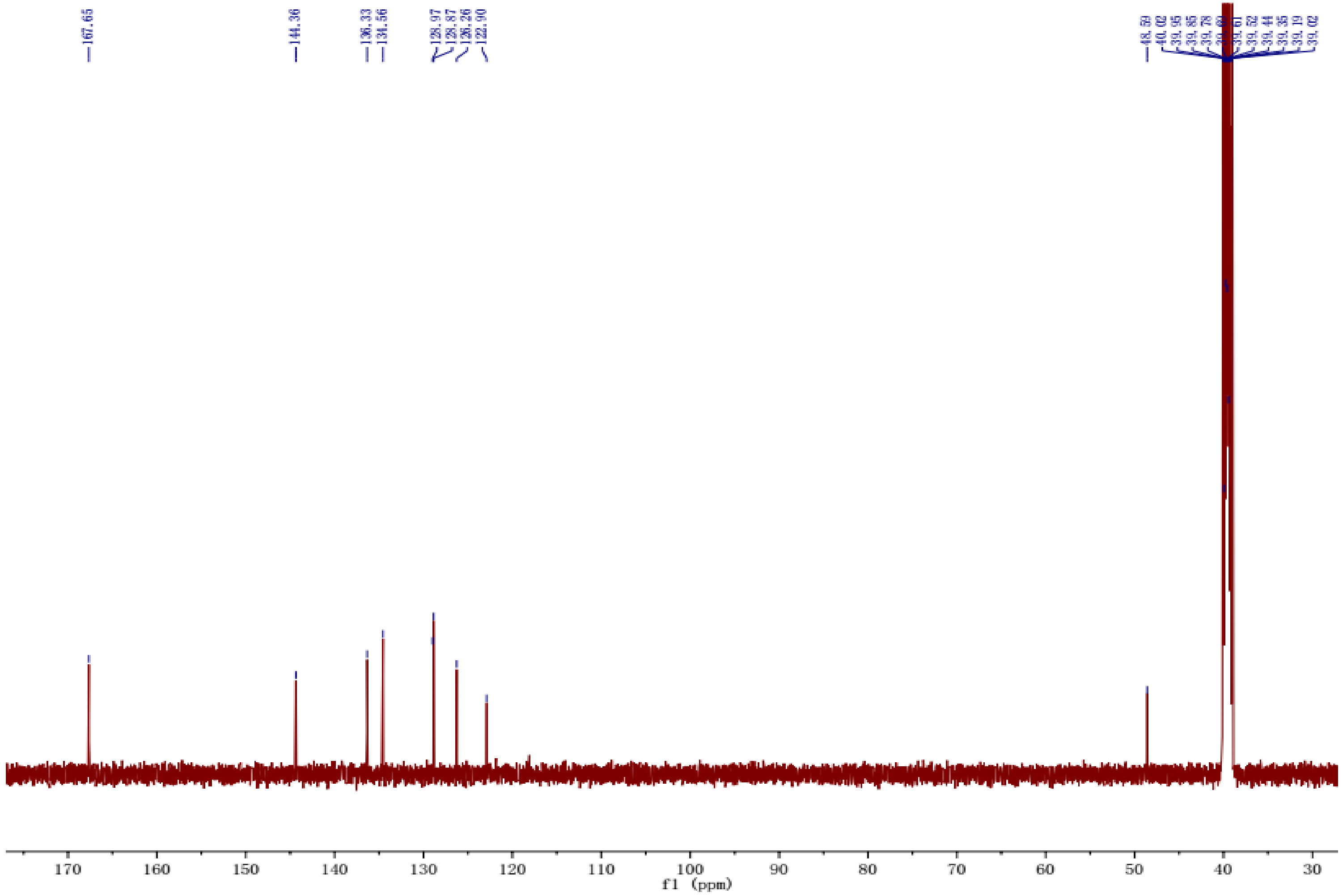

Figure S8. HMQC spectrum (500 MHz, DMSO-d_6) of **2**.

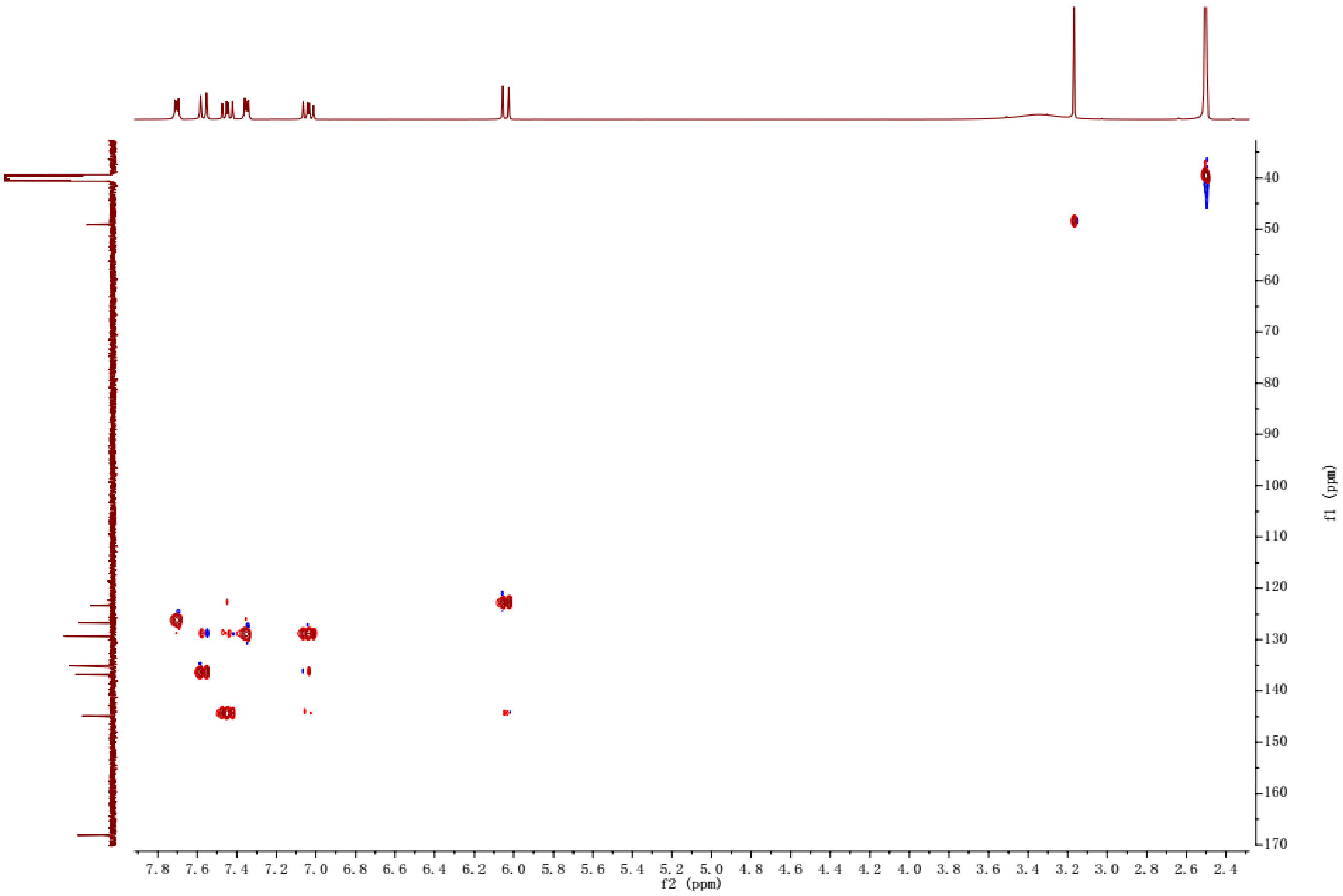

Figure S9. HMBC spectrum (500 MHz, DMSO-d_6) of **2**.

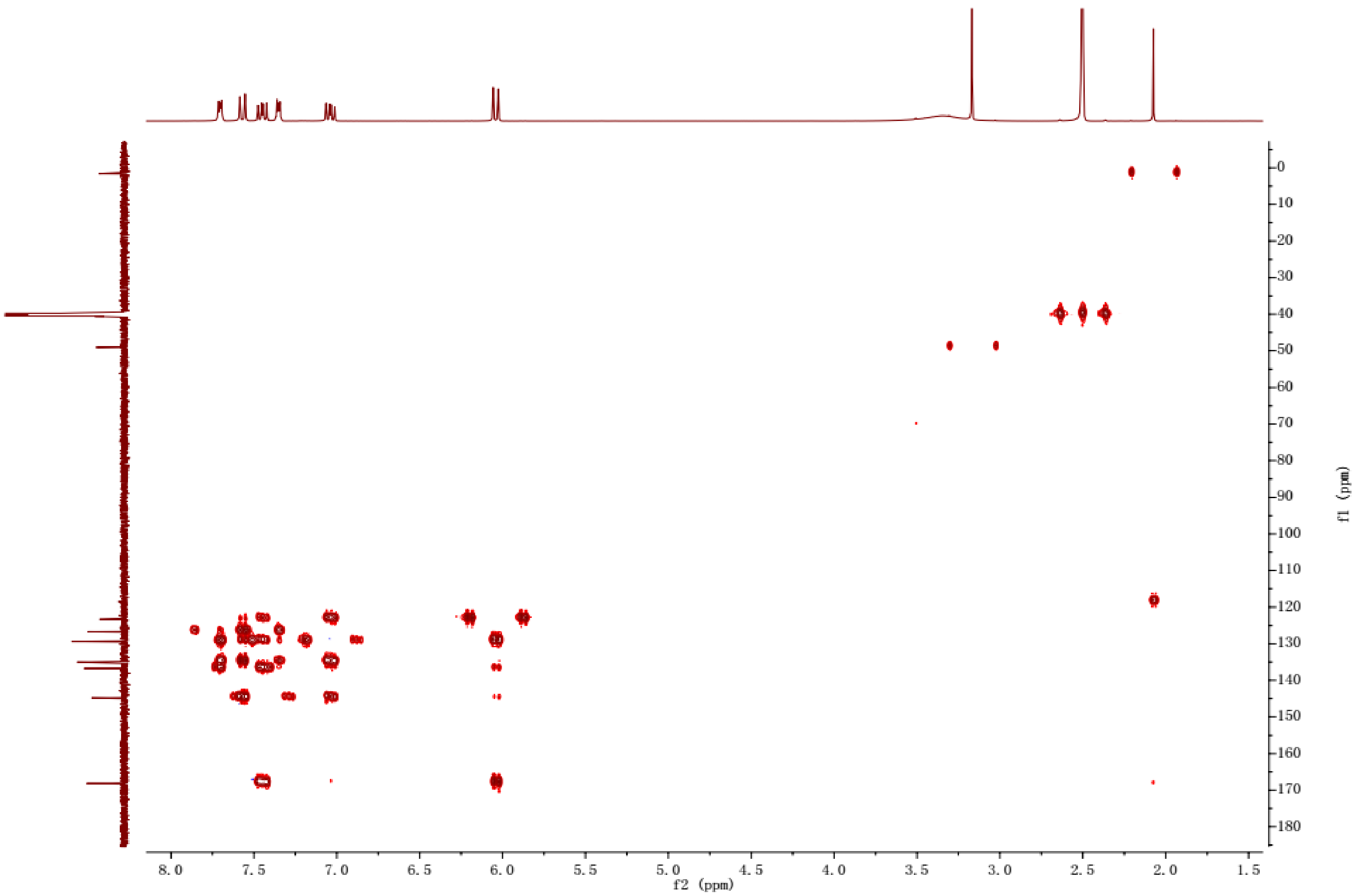

Figure S10. HR-ESI-MS of **2**.

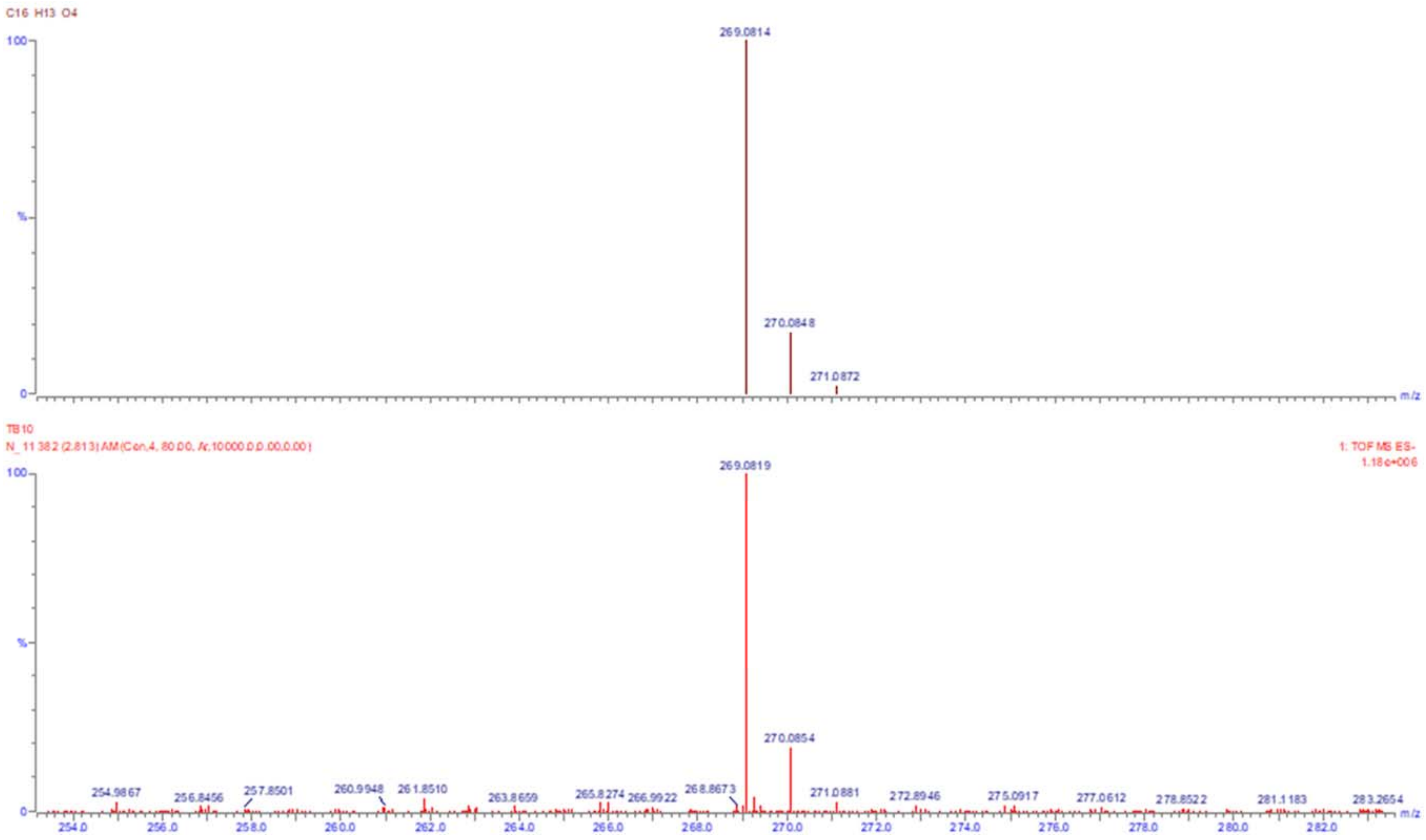

Figure S11. IR spectrum of **2.**

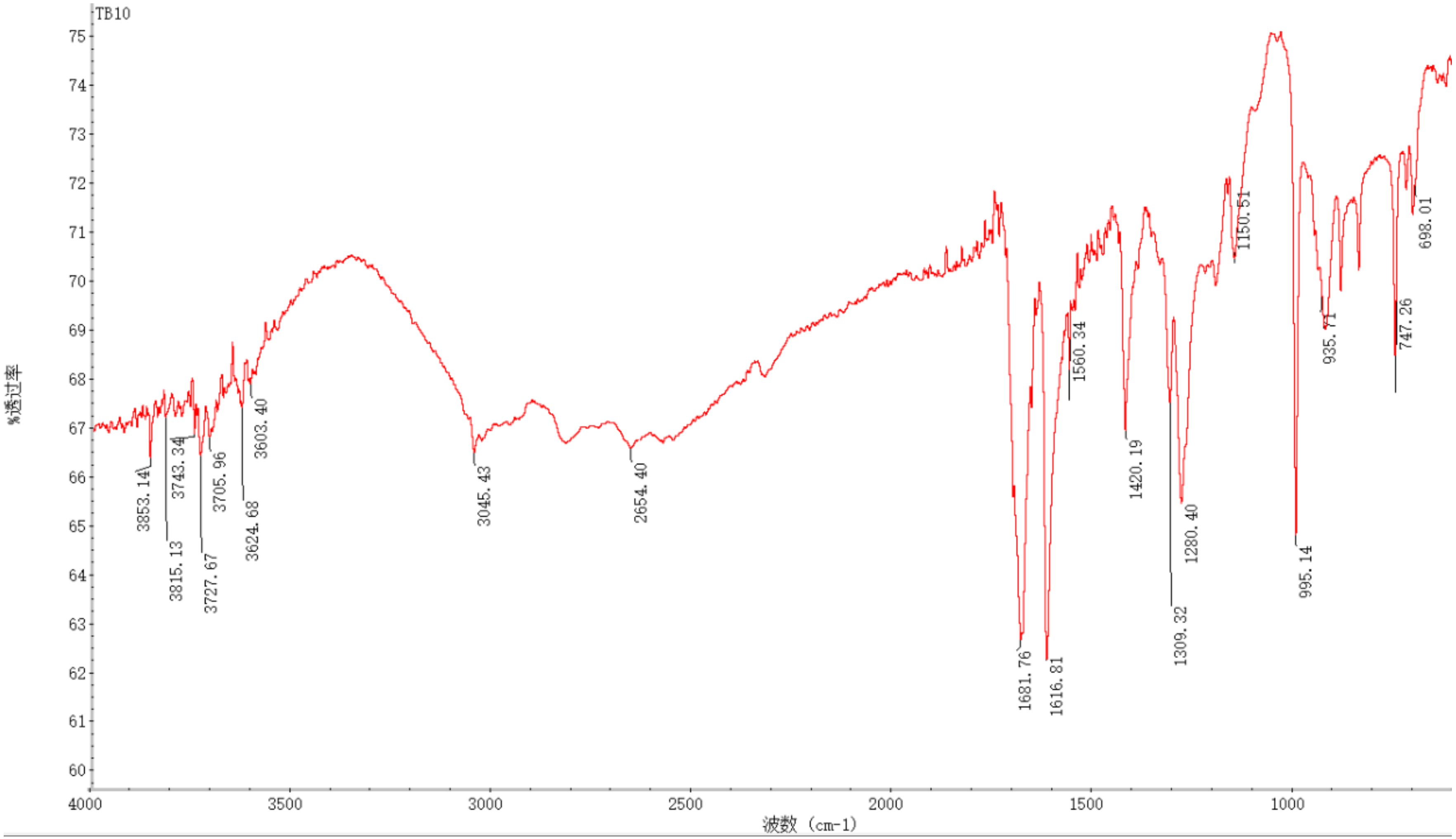

Figure S12. ^{1}H-NMR spectrum (500 MHz, DMSO-d_6) of **3**.

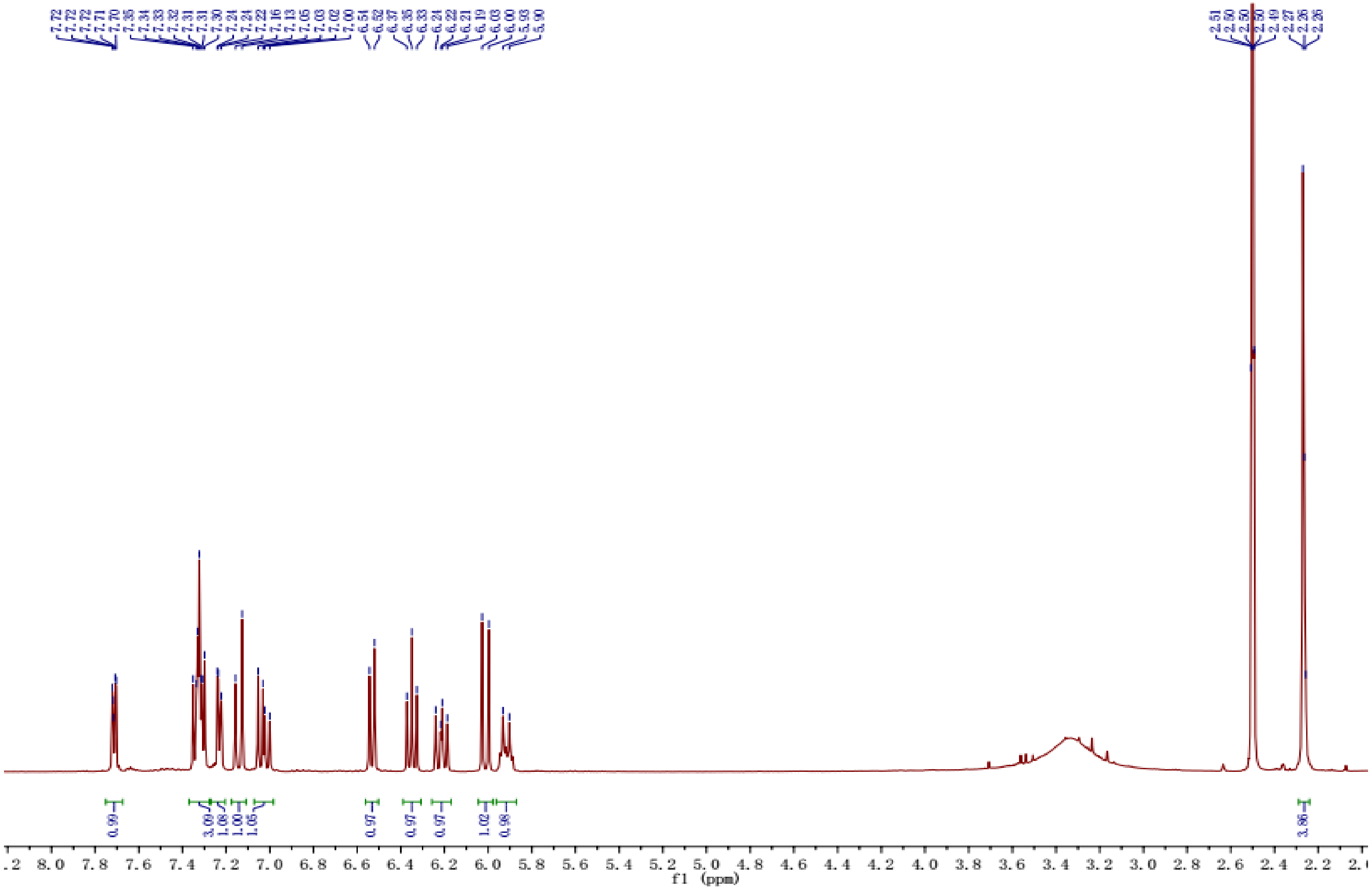

Figure S13. ^{13}C-NMR spectrum (125 MHz, DMSO-*d*$_6$) of **3**.

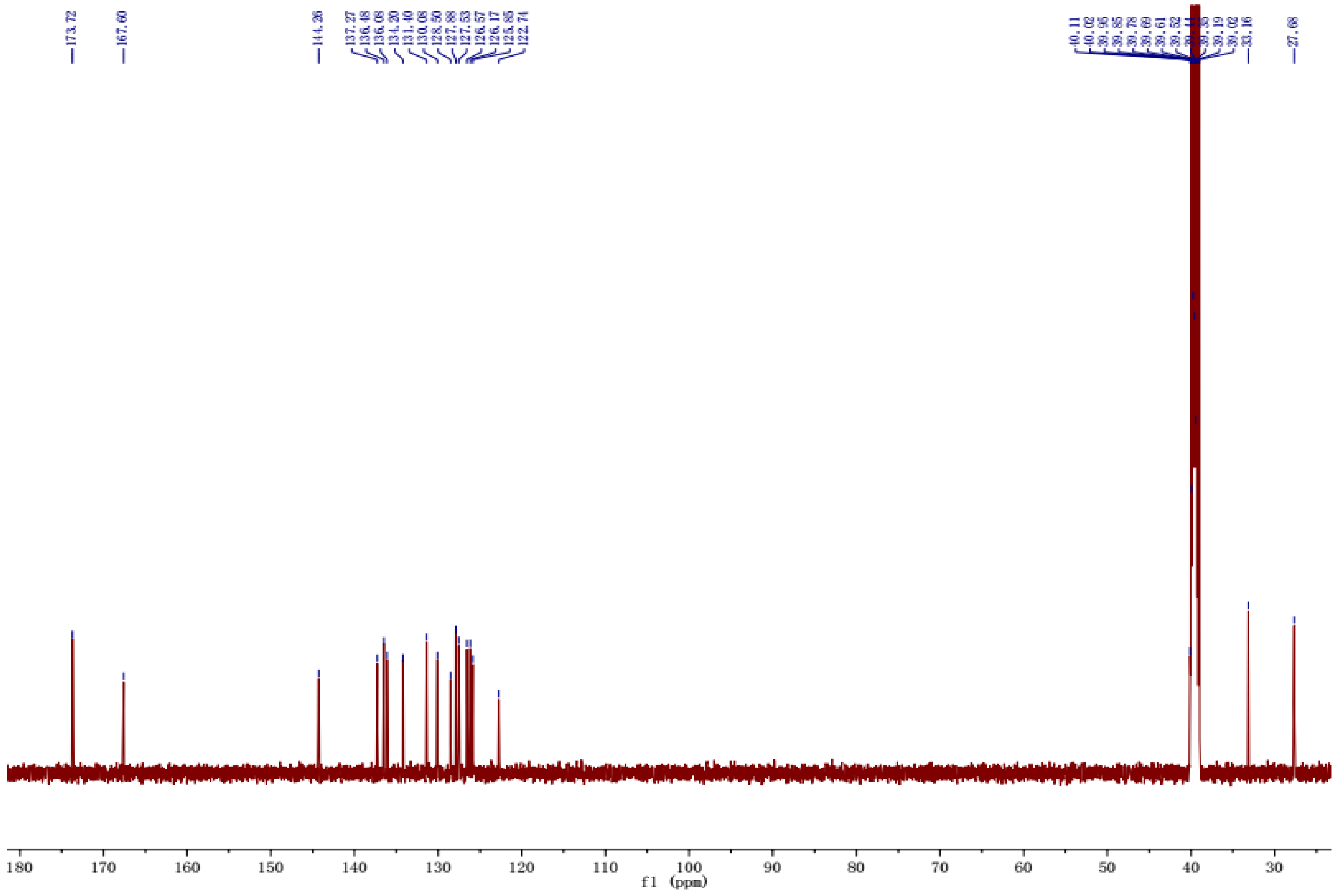

Figure S14. HMBC spectrum (500 MHz, DMSO-d_6) of **3**.

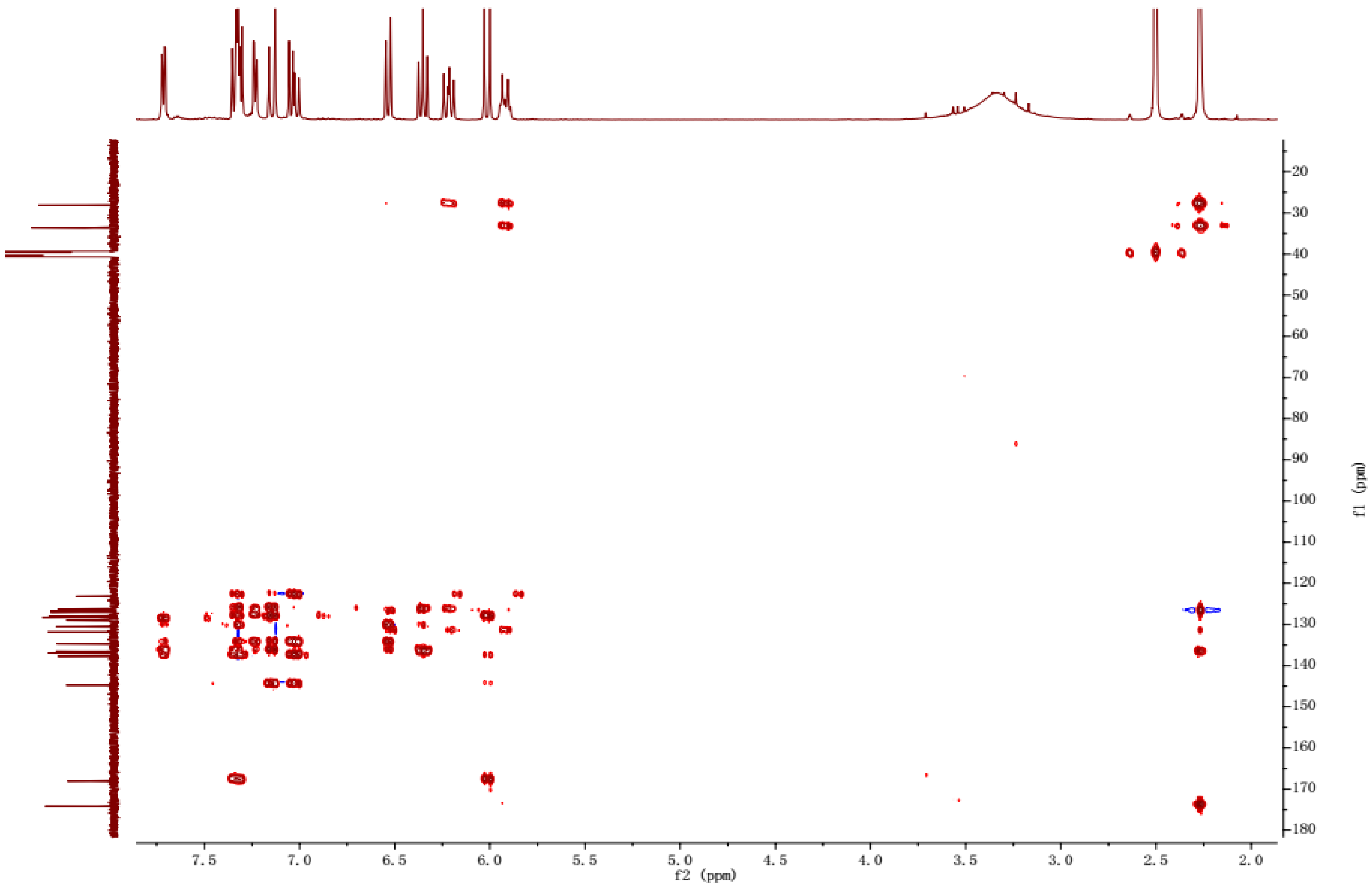

Figure S15. HR-ESI-MS of **3**.

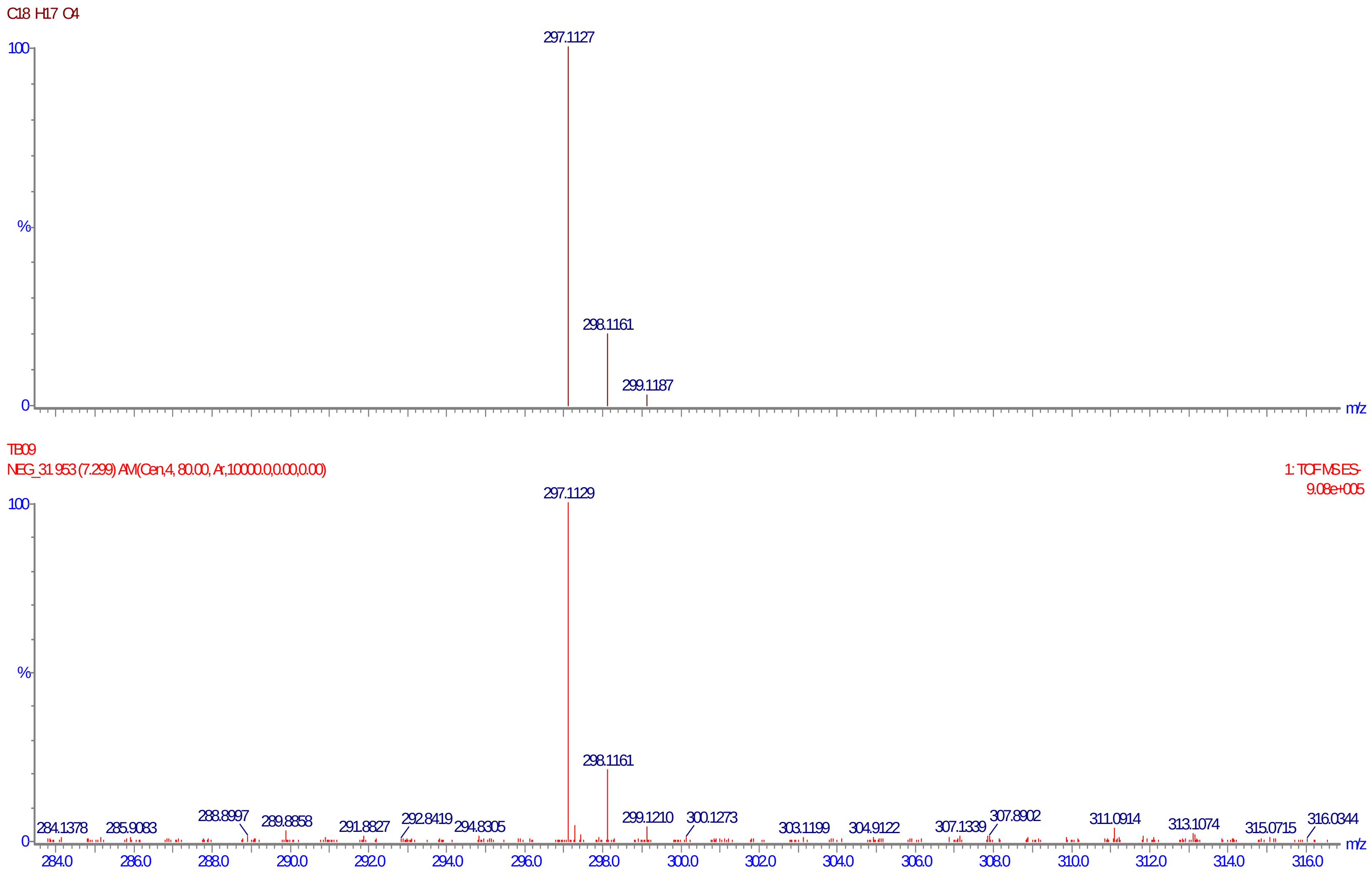

Figure S16. IR spectrum of **3.**

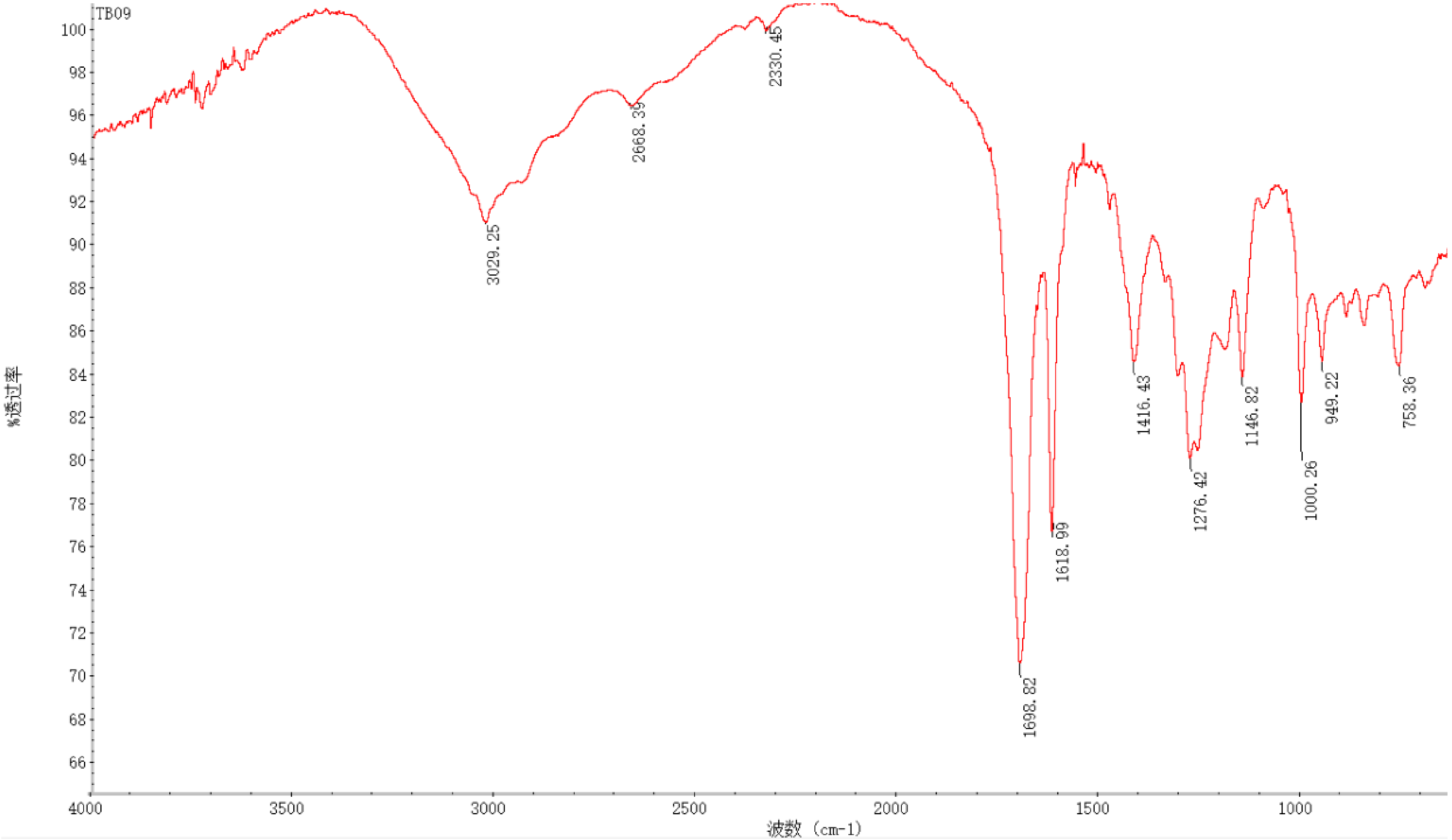

Figure S17. ^{1}H-NMR spectrum (500 MHz, DMSO-d_6) of **4**.

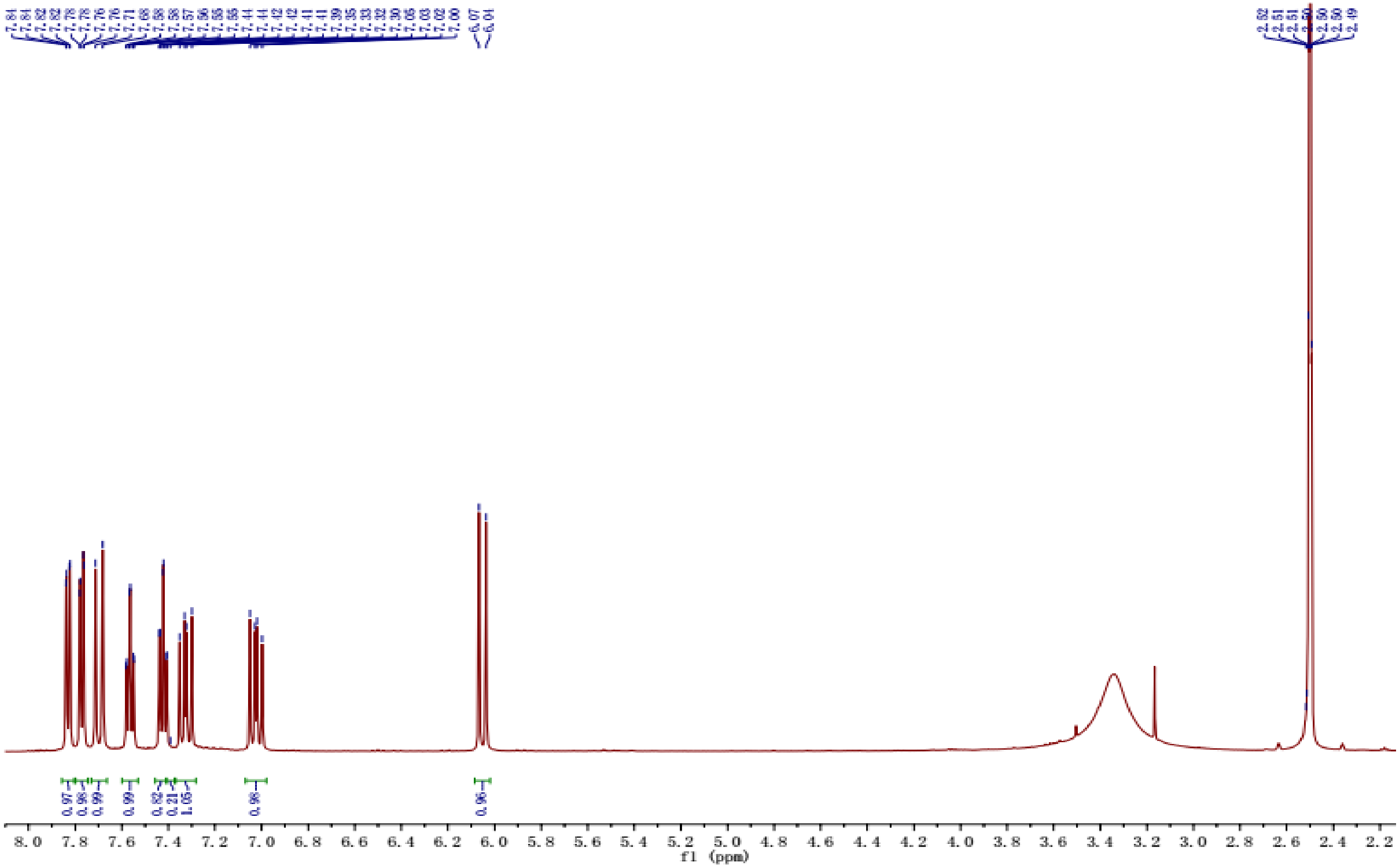

Figure S18. ^{13}C-NMR spectrum (125 MHz, DMSO-d_6) of **4**

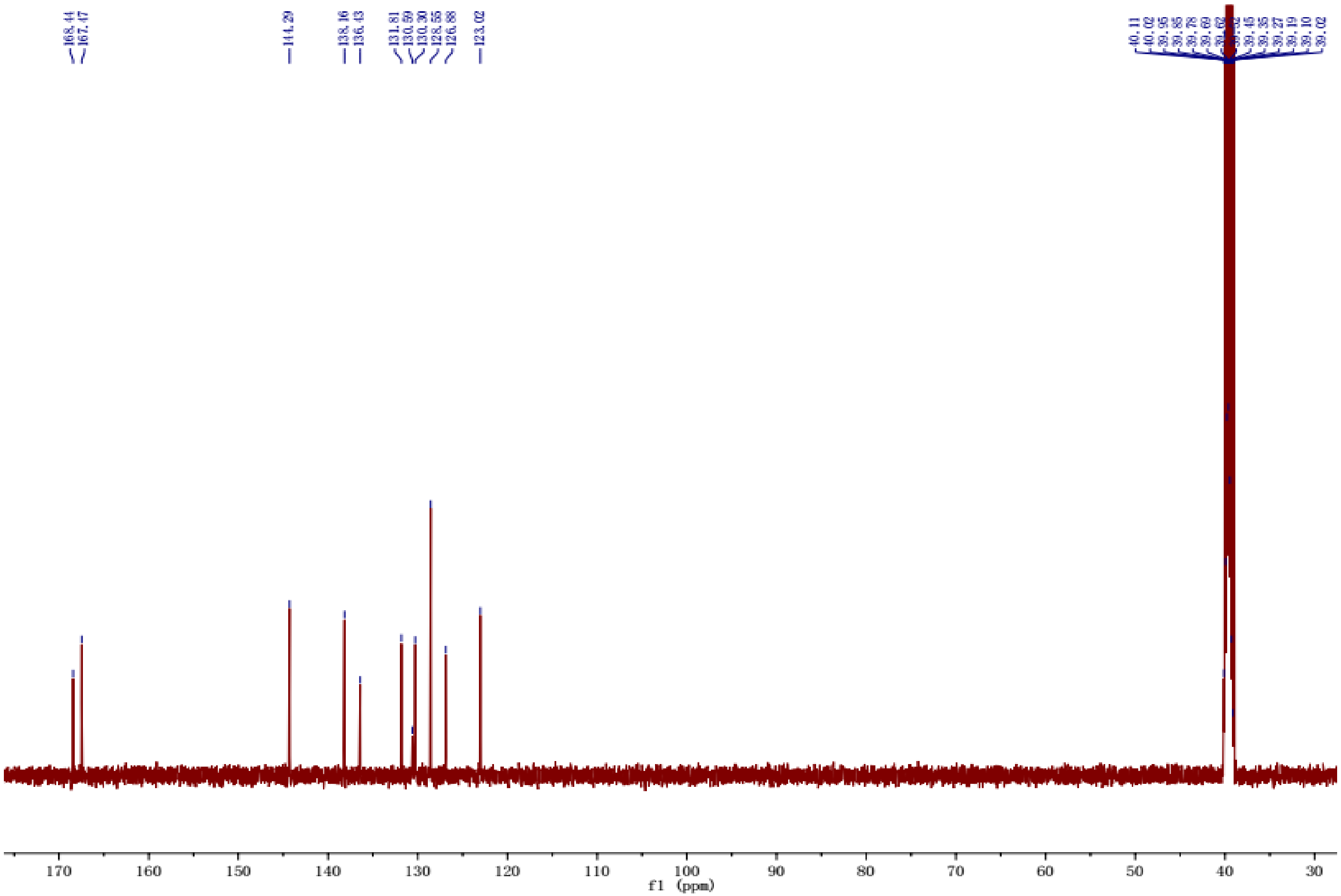

Figure S19. HMBC spectrum (500 MHz, DMSO-d_6) of **4**.

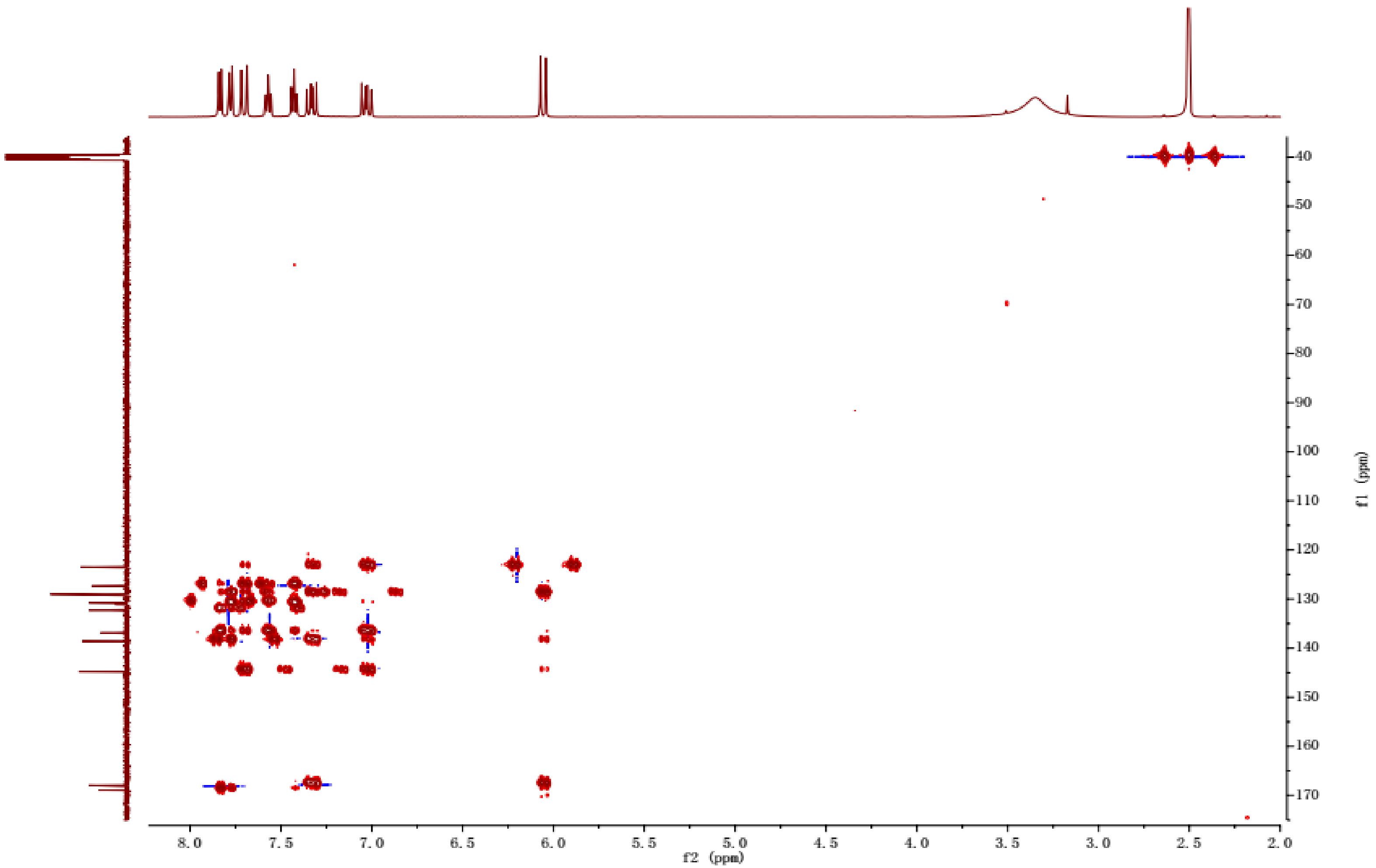

Figure S20. HR-ESI-MS of **4**.

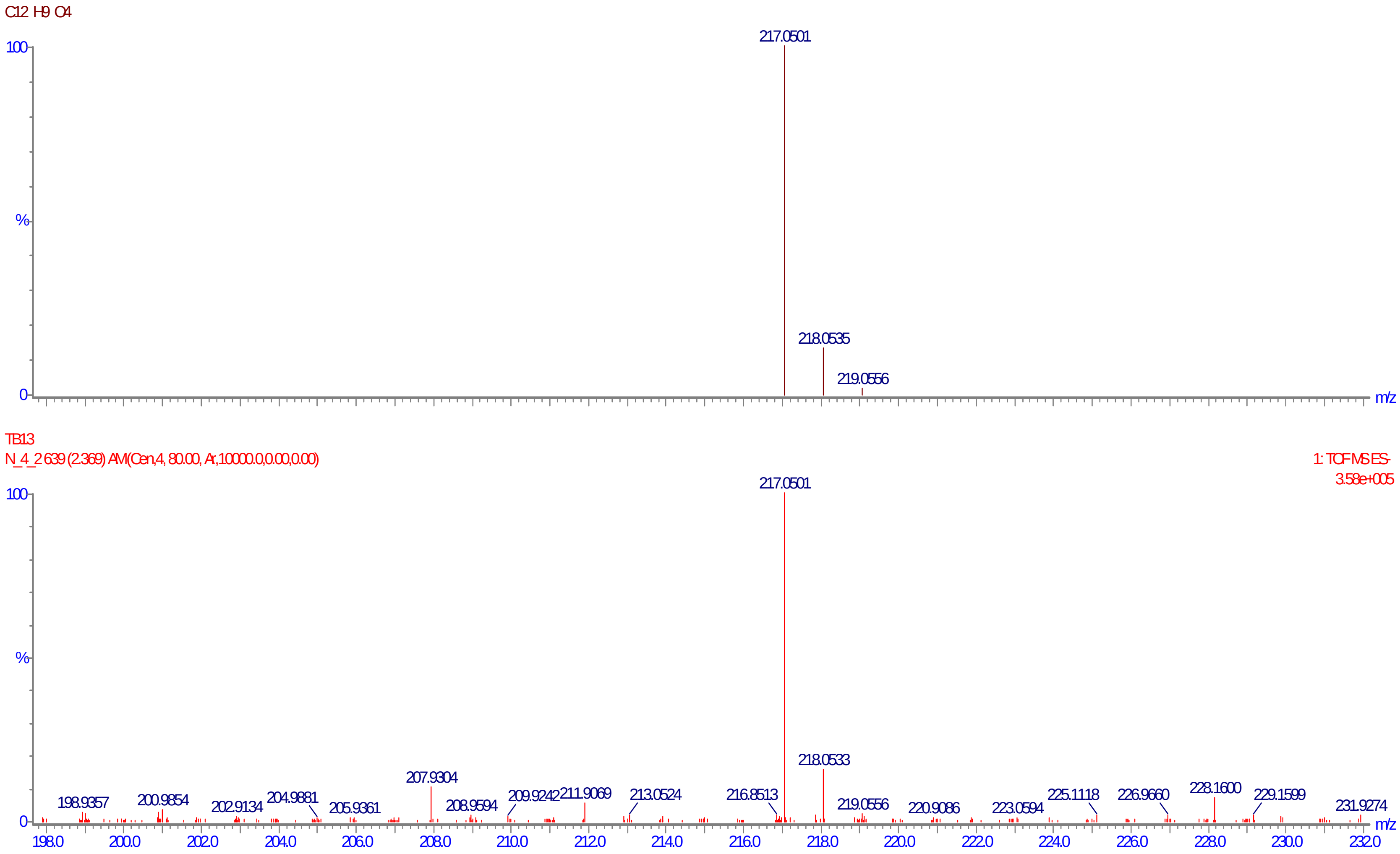

Figure S21. IR spectrum of **4.**

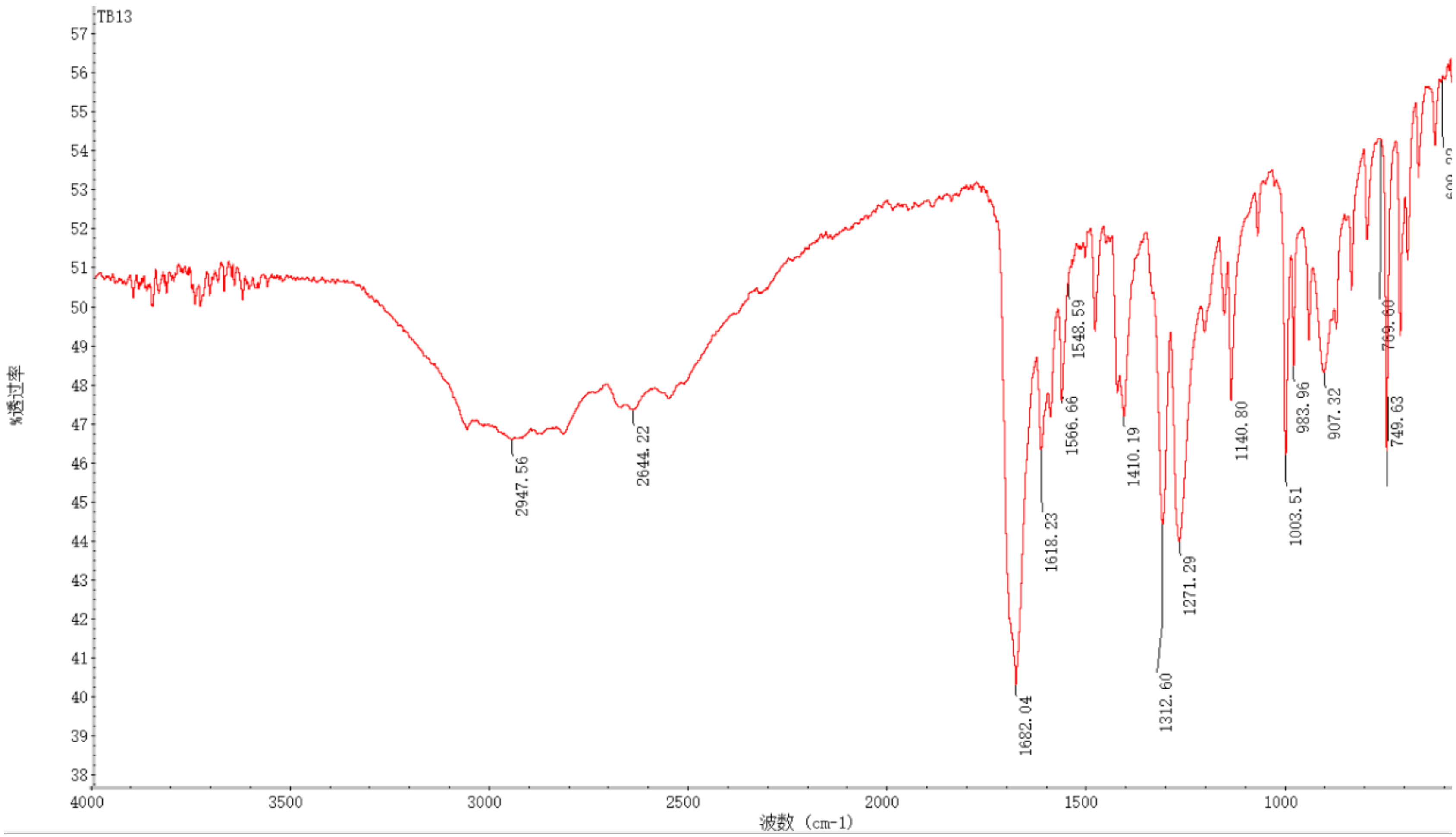

Figure S22. ^{1}H-NMR spectrum (500 MHz, DMSO-d_6) of **5**.

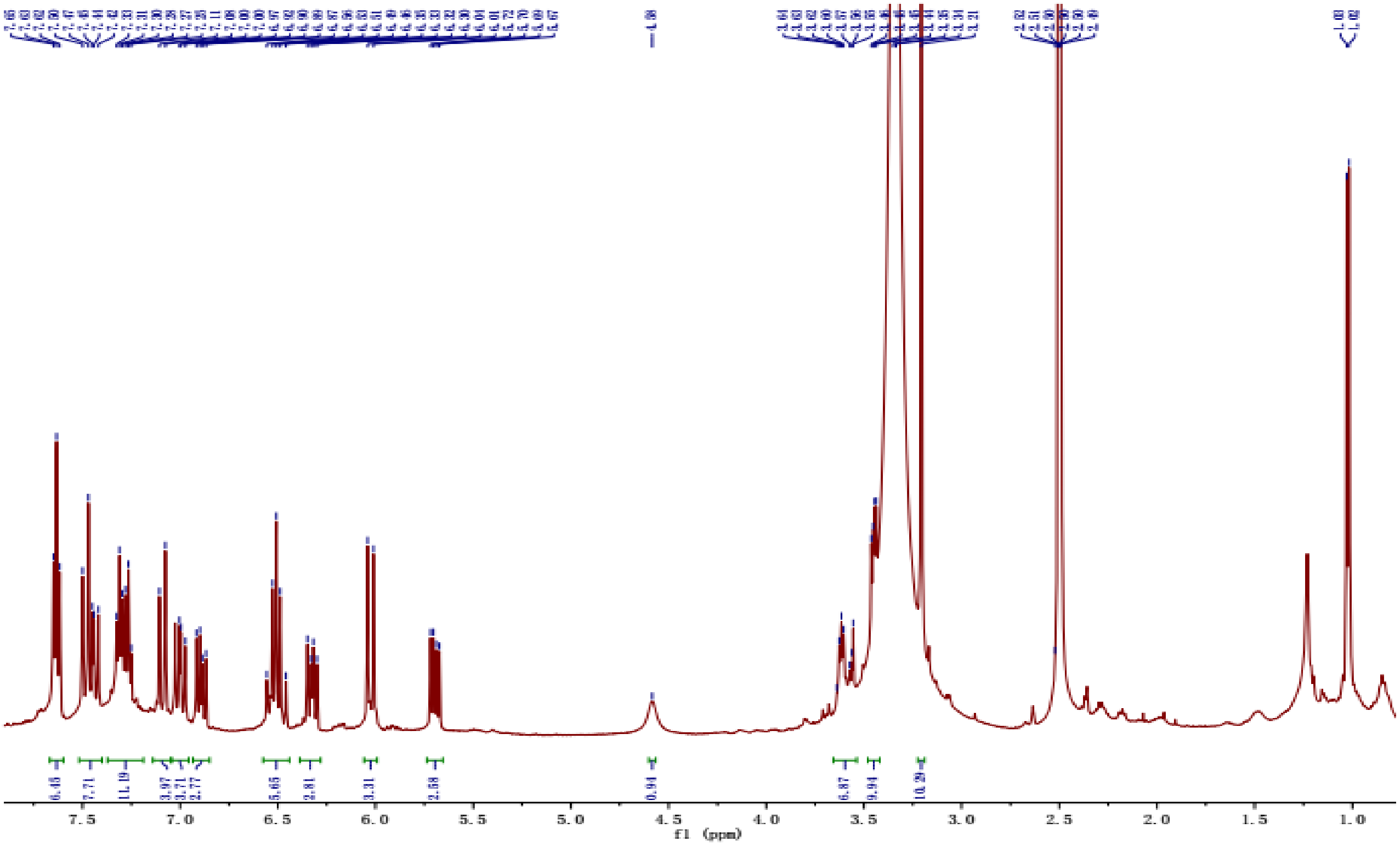

Figure S23. ^{13}C-NMR spectrum (125 MHz, DMSO-d_6) of **5**.

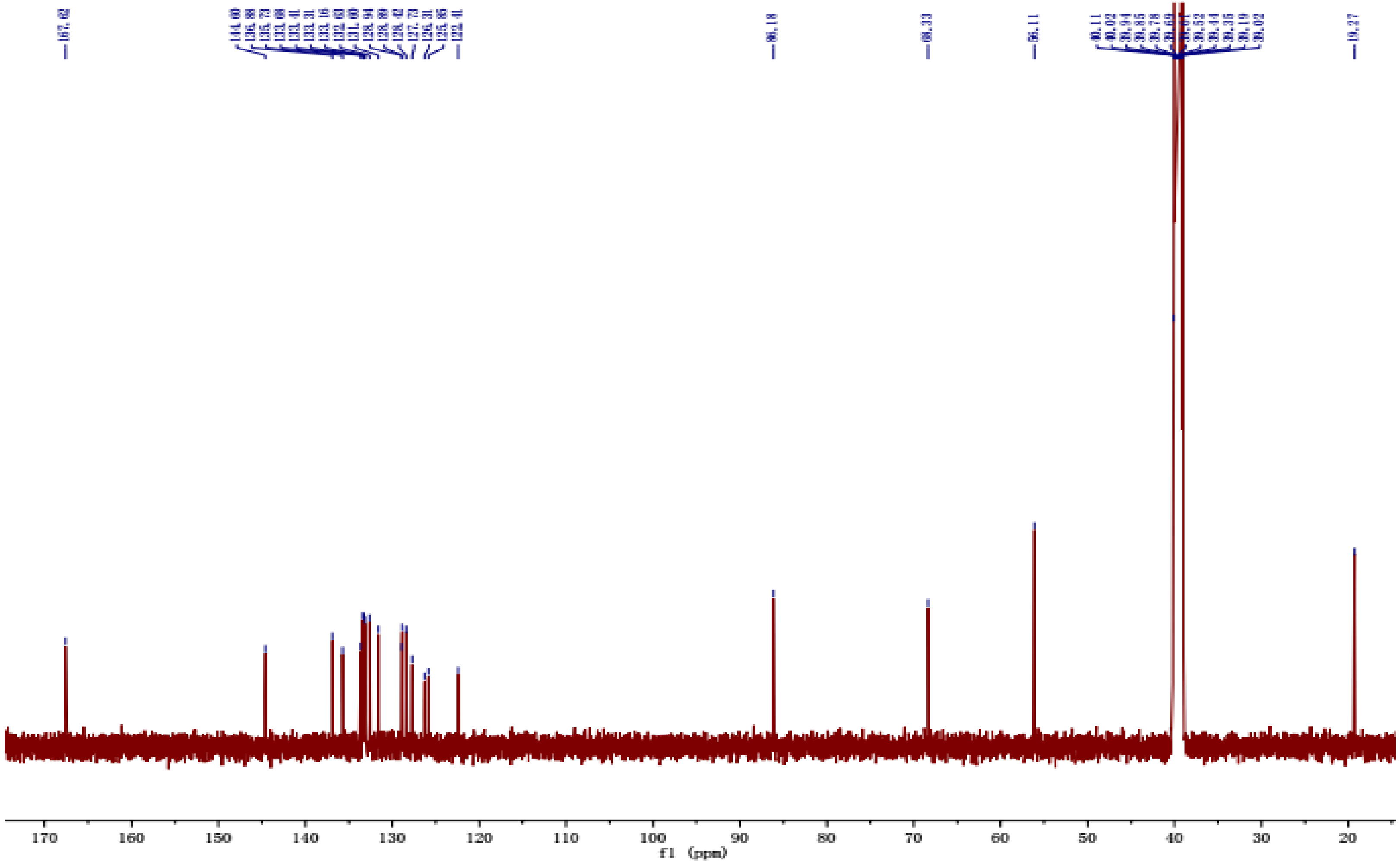

Figure S24. ^{1}H-^{1}H COSY spectrum (500 MHz, DMSO-d_6) of **5**.

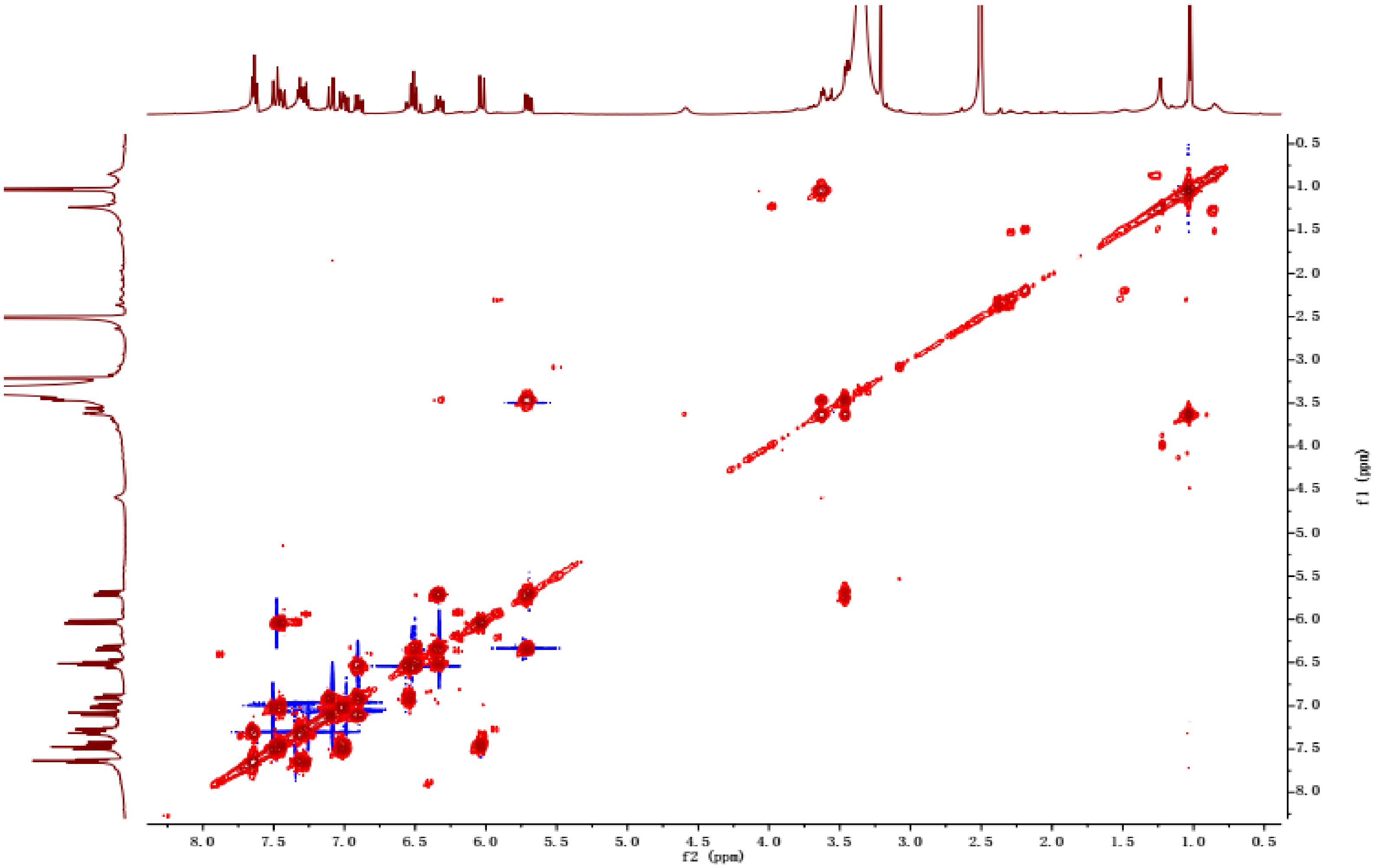

Figure S25. HMBC spectrum (500 MHz, DMSO-d_6) of **5**.

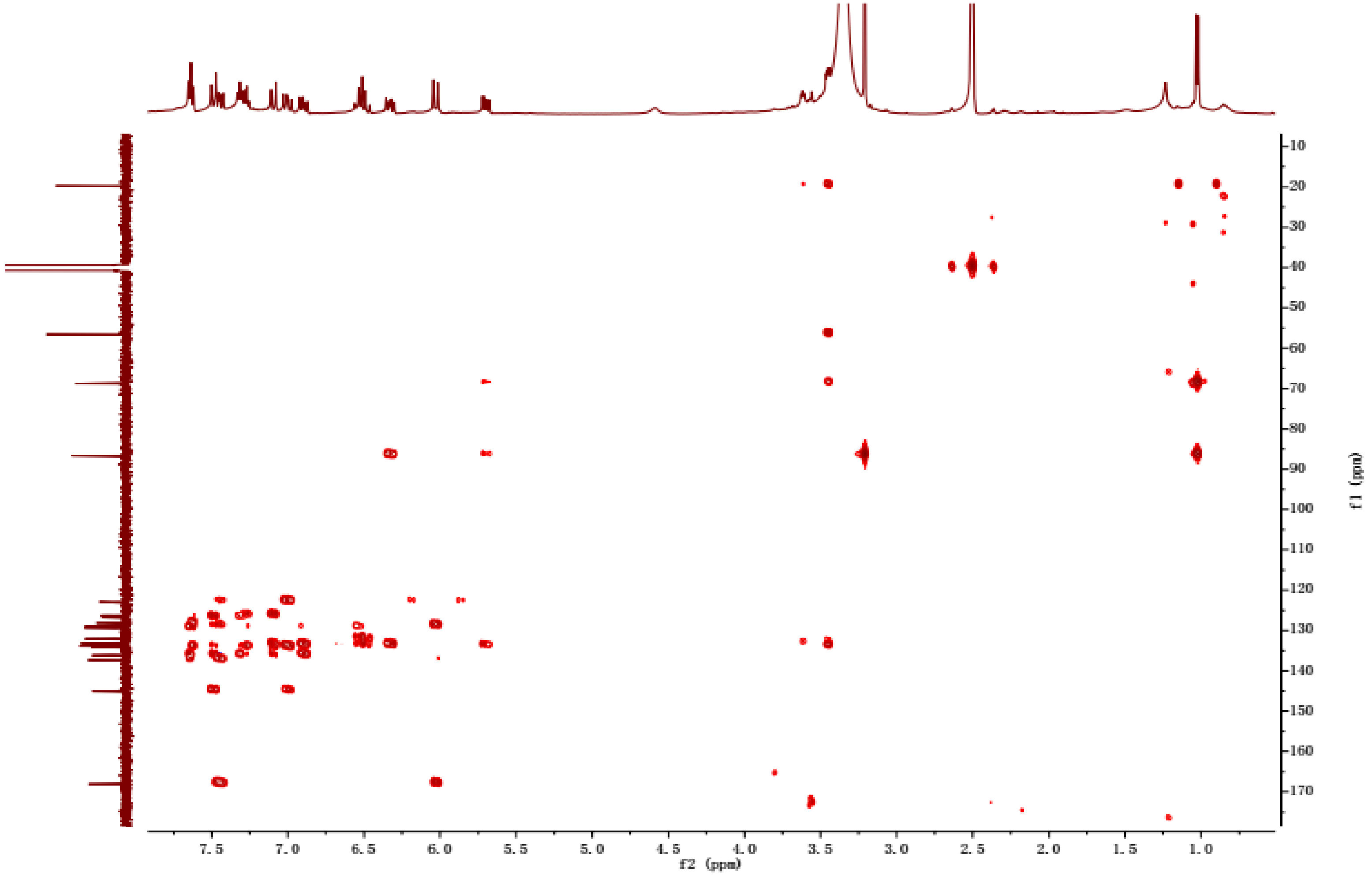

Figure S26. HR-ESI-MS of **5**.

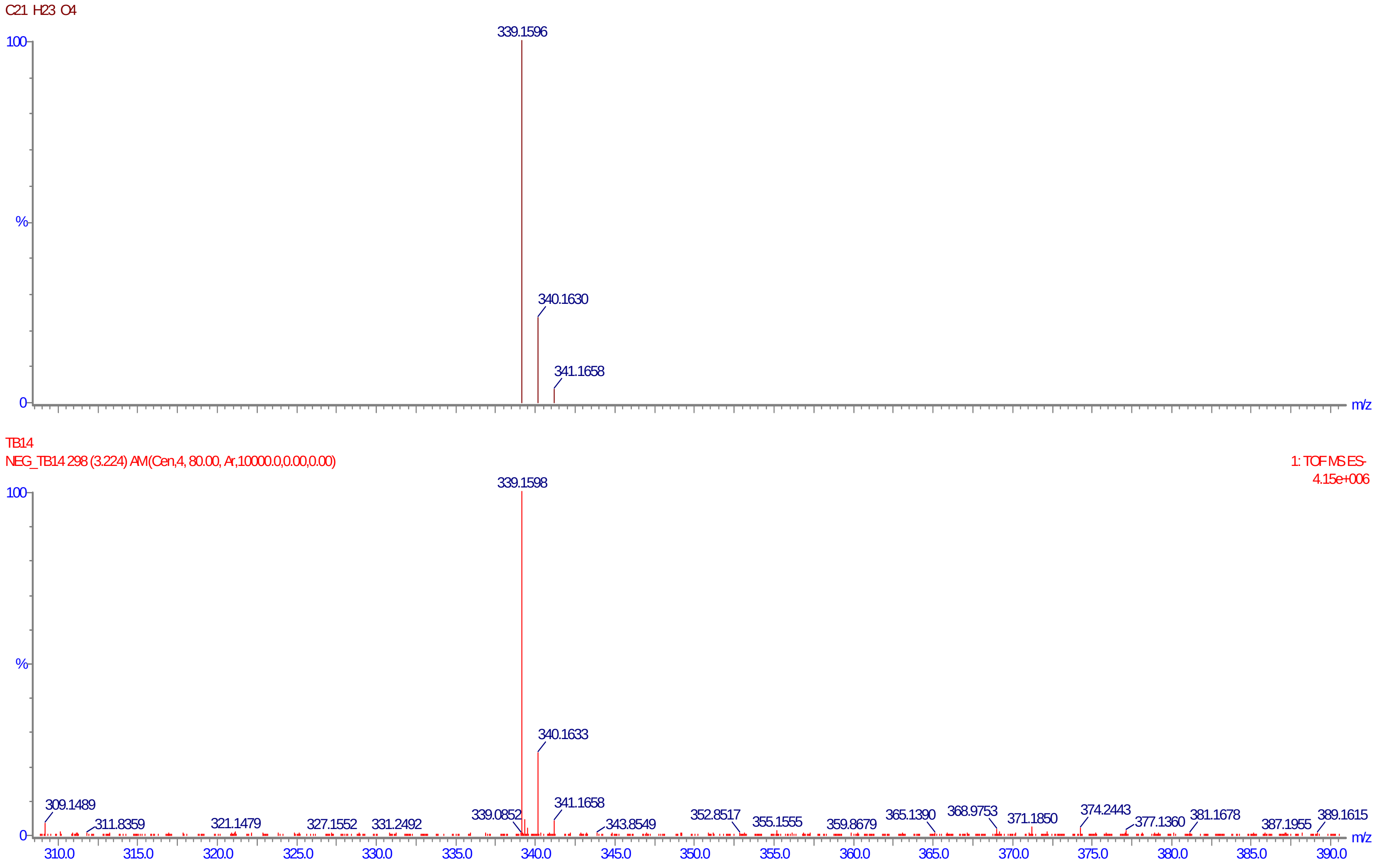

Figure S27. IR spectrum of **5.**

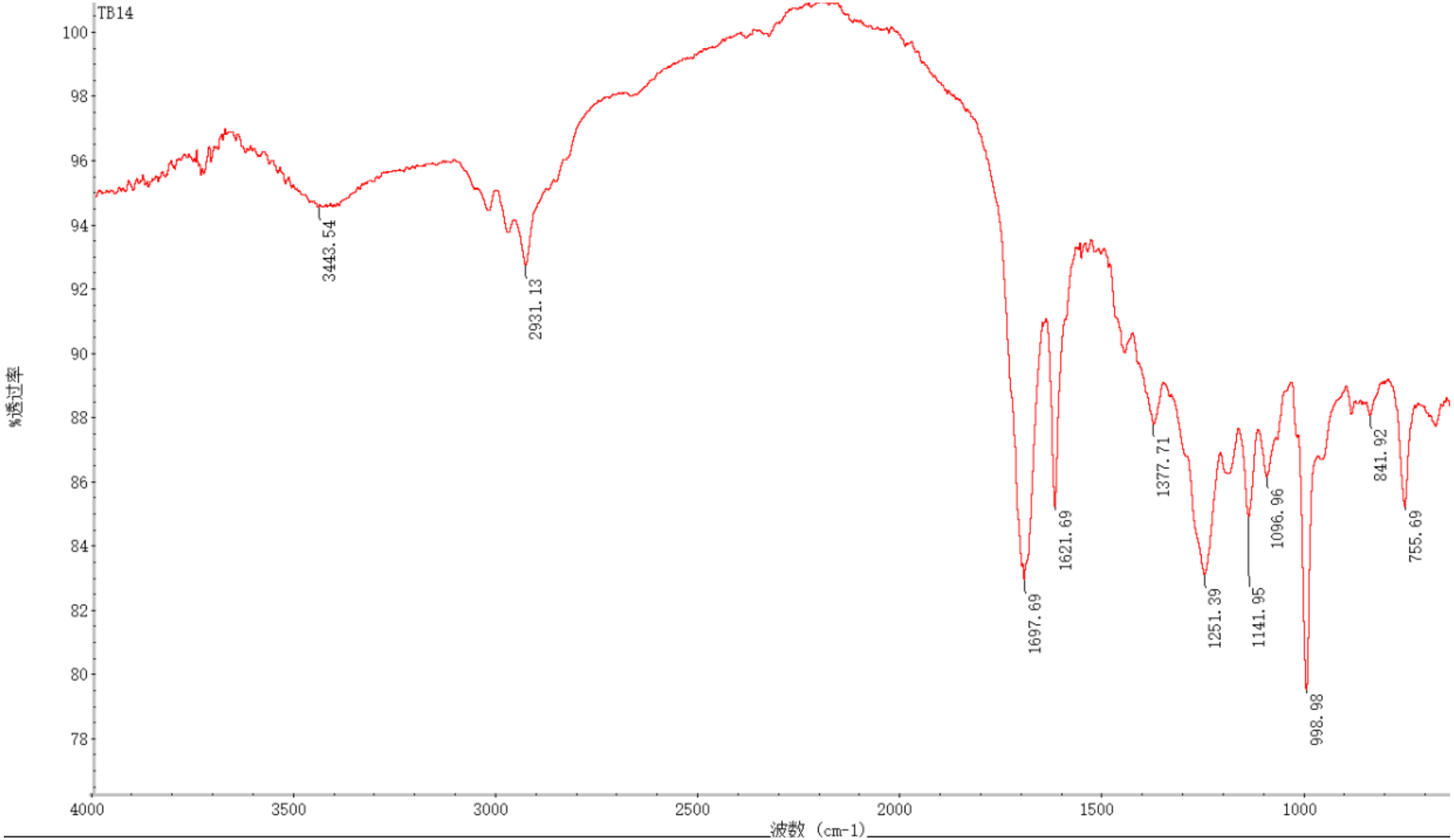

Figure S28. ^{1}H-NMR spectrum (500 MHz, DMSO-d_6) of **6**.

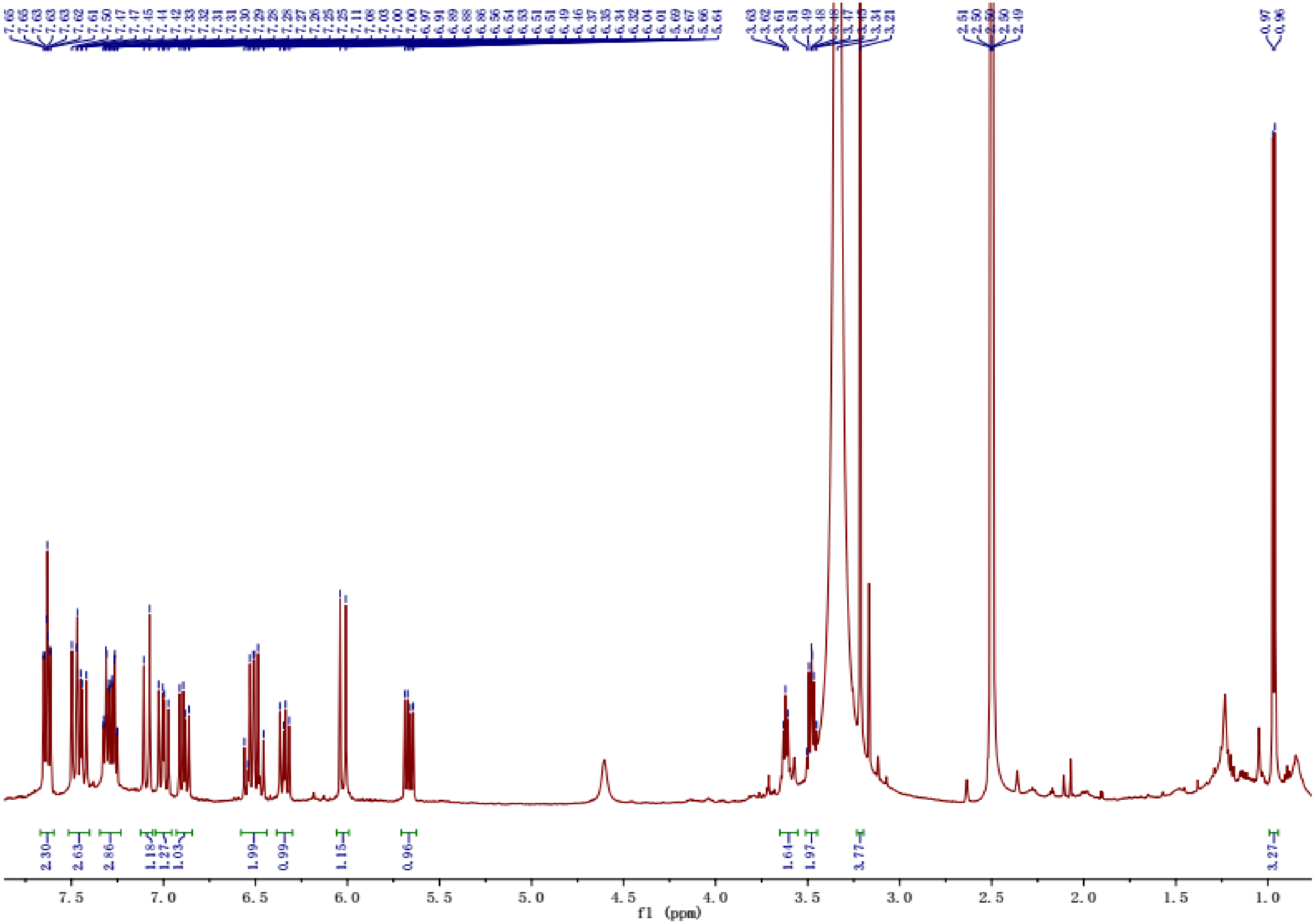

Figure S29. ^{13}C-NMR spectrum (125 MHz, DMSO-d_6) of **6**.

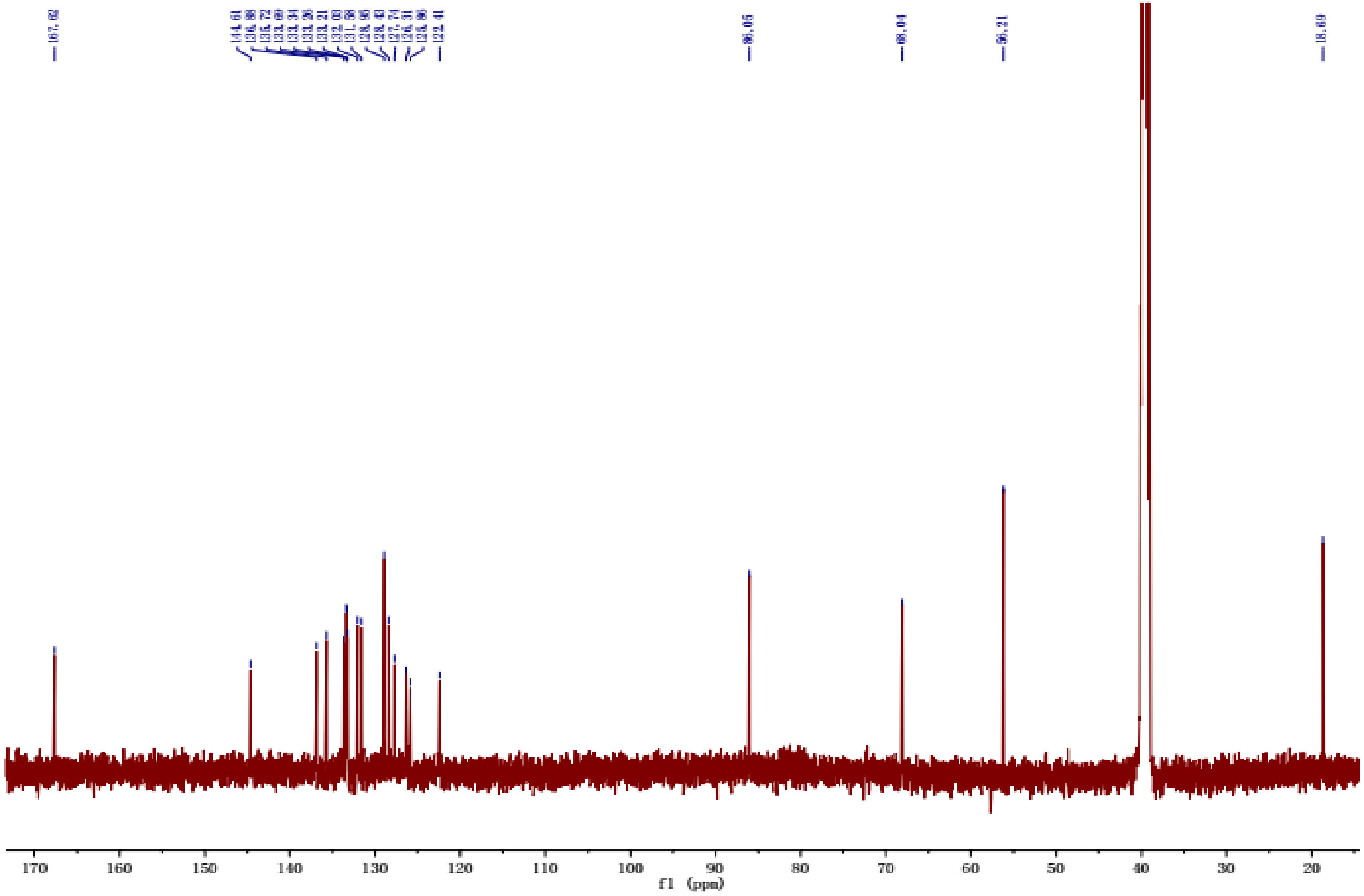

Figure S30. ^{1}H-^{1}H COSY spectrum (500 MHz, DMSO-d_6) of **6**.

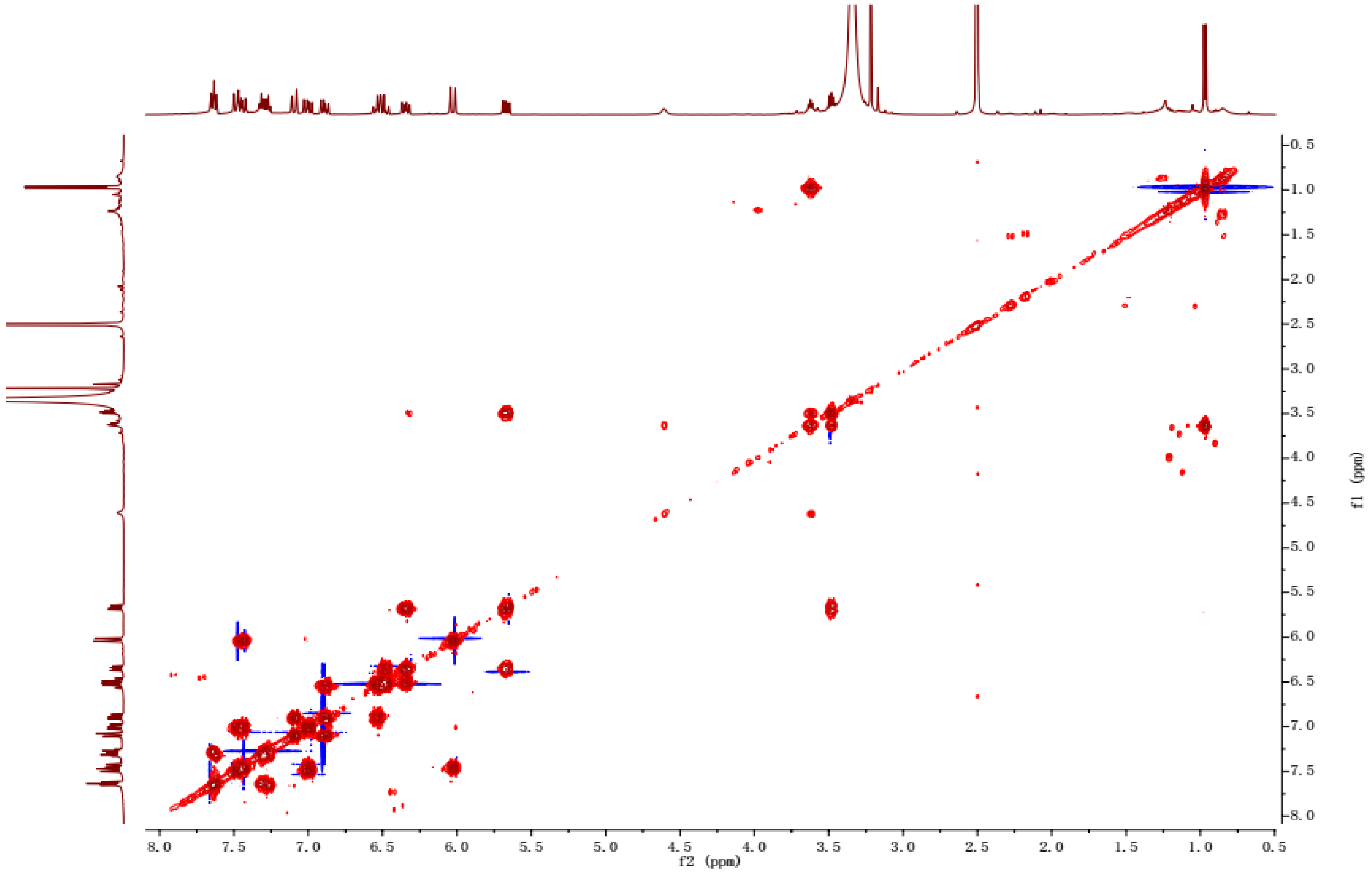

Figure S31. HMBC spectrum (500 MHz, DMSO-d_6) of **6**.

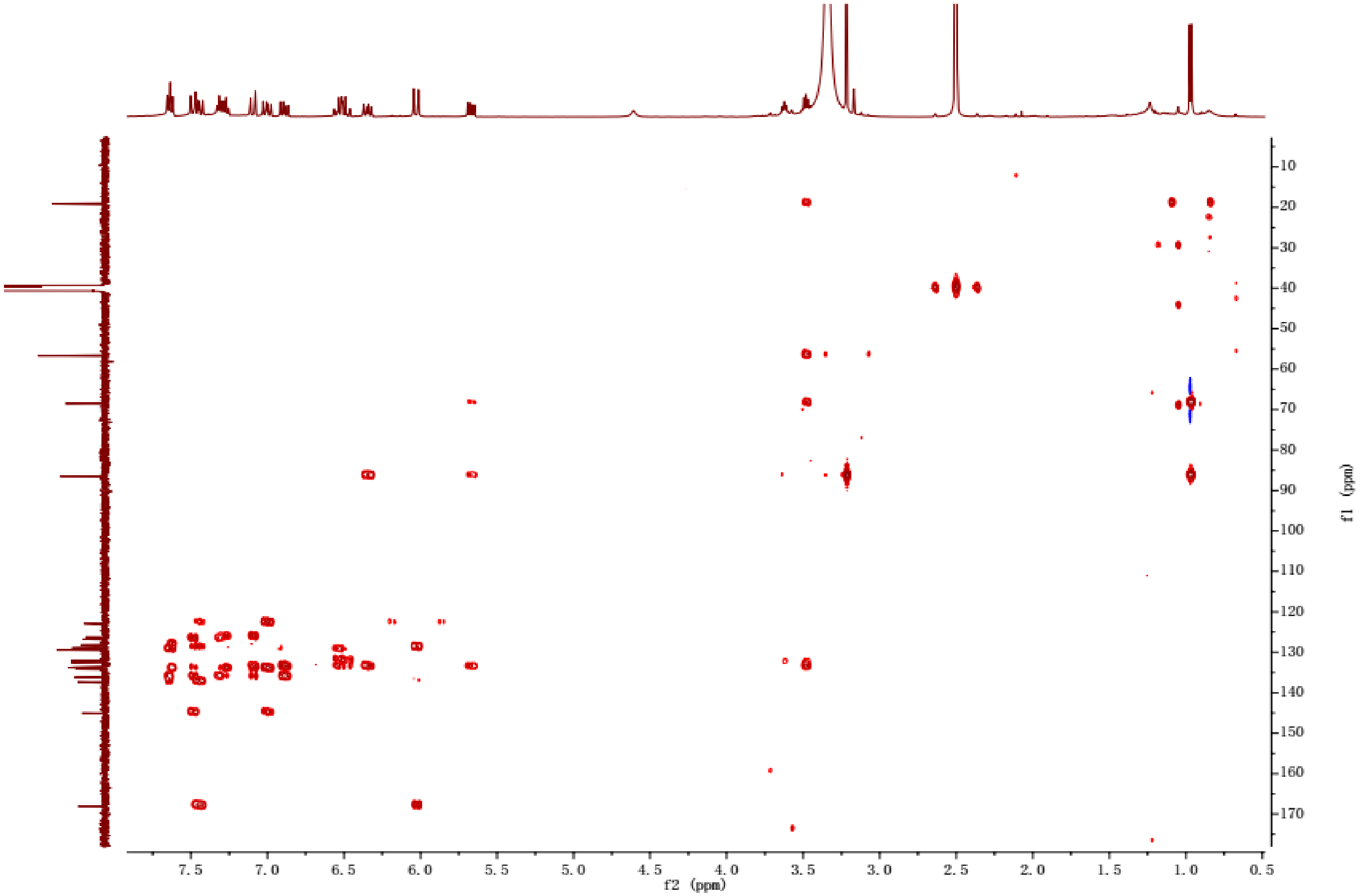

Figure S32. HR-ESI-MS of **6**.

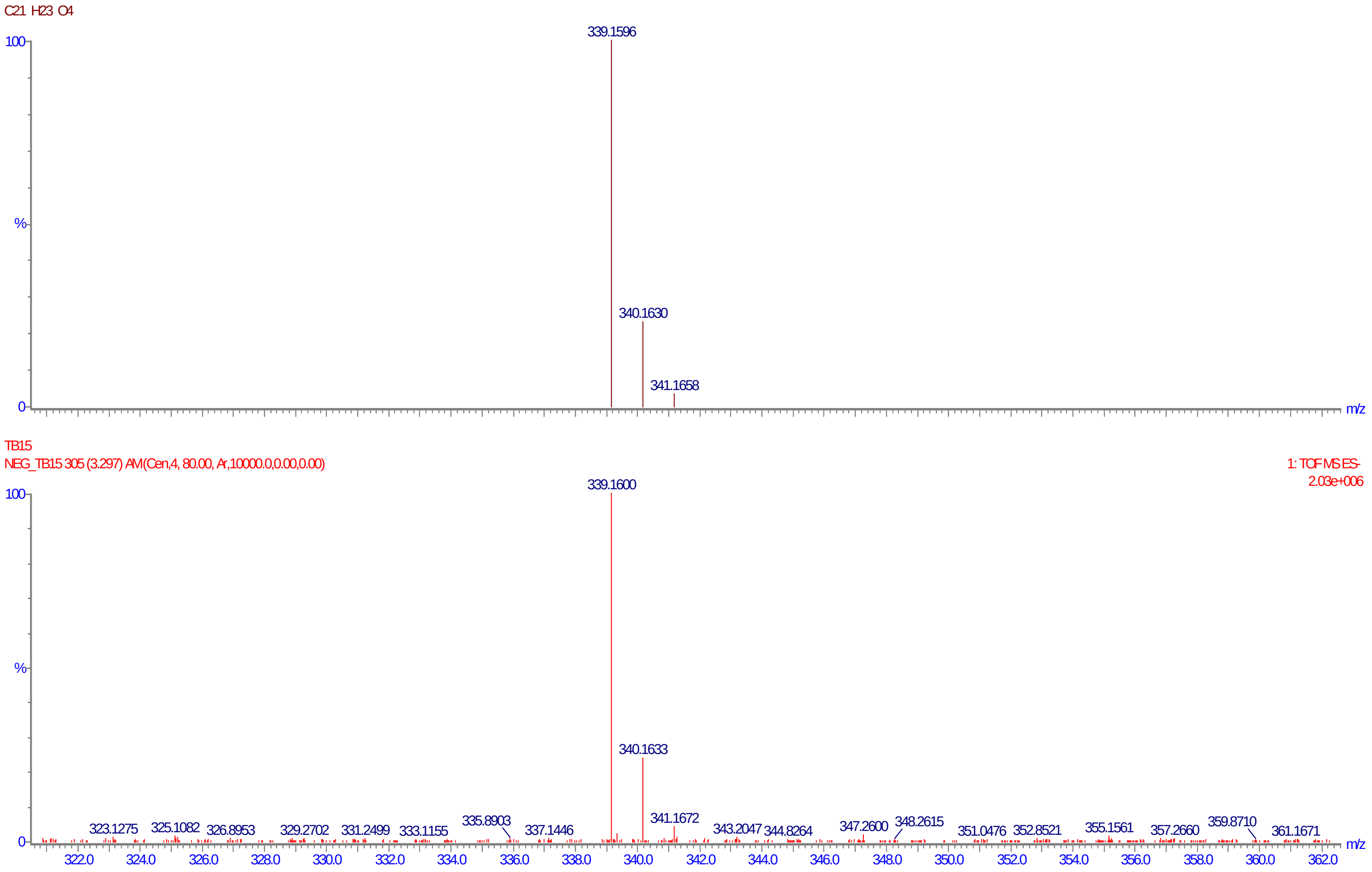

Figure S33. IR spectrum of **6.**

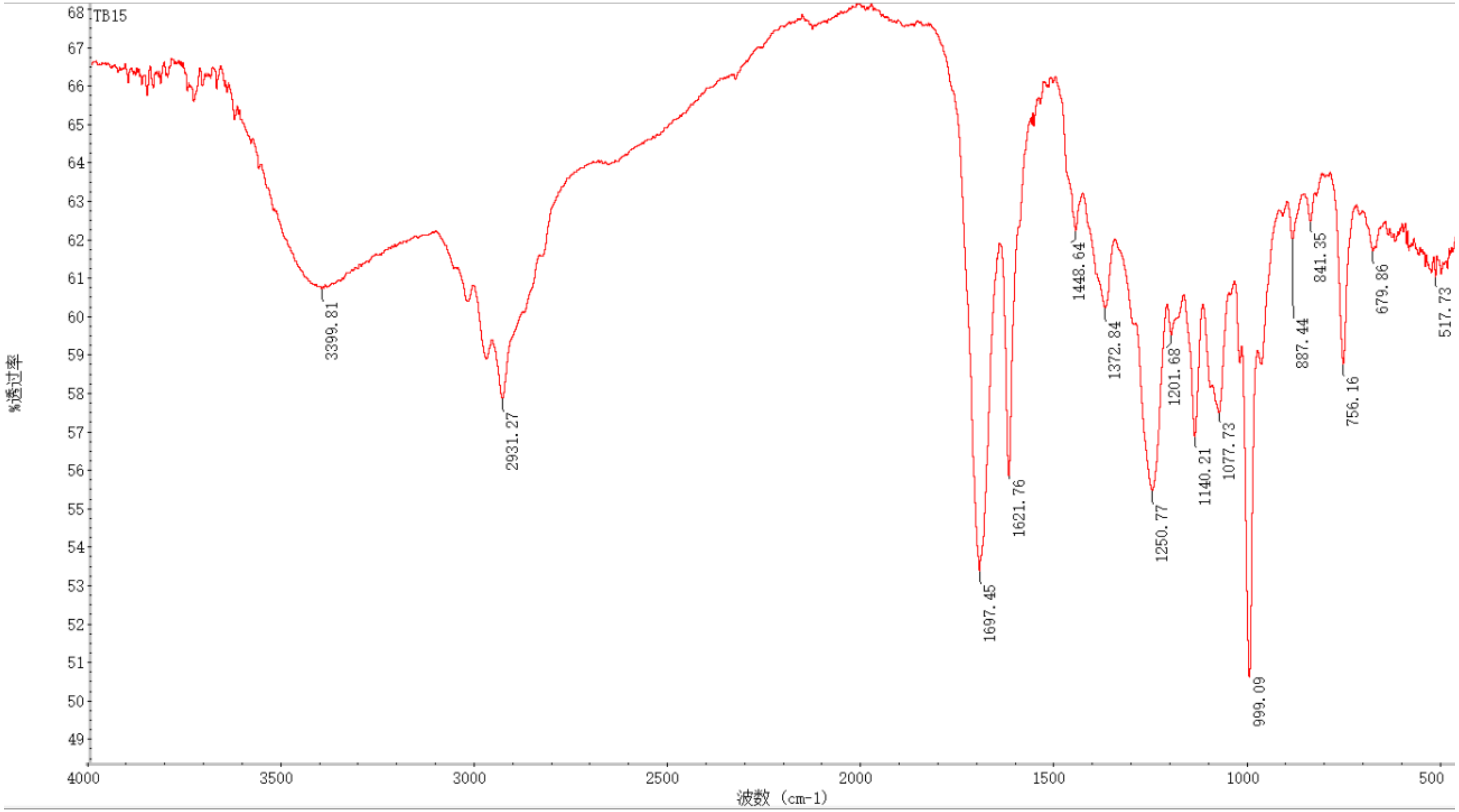

Figure S34. Experimental chemical shifts and calculated shielding tensors for PD4+ probability analysis for compound **5**. (isomer **5a**: rel-(18*S*,19*S*)-**5**; isomer **5b**: rel-(18*S*,19*R*)-**5**).

Functional		Solvent?		Basis Set		Type of Data	
B3LYP		PCM		6-31G(d)		Shielding Tensors	
		DP4+	99.91%	0.09%	–	–	–
Nuclei	sp2?	xperiments	Isomer 1	Isomer 2	Isomer 3	Isomer 4	Isomer 5
C	x	167.6	28.7036	28.632			
C	x	122.4	74.762	74.9155			
C	x	144.6	50.9766	50.8417			
C	x	128.4	65.0218	65.1043			
C	x	136.9	54.051	53.9157			
C	x	133.7	59.3529	59.3343			
C	x	126.3	66.7804	66.7639			
C	x	127.7	68.7908	68.7505			
C	x	128.9	67.4192	67.4096			
C	x	125.8	67.3327	67.2188			
C	x	135.7	55.3389	55.3375			
C	x	128.9	58.1421	57.8932			
C	x	131.6	66.7366	66.6921			
C	x	133.4	65.0501	64.7953			
C	x	133.3	65.9629	65.9584			
C	x	133.2	69.5645	67.6536			
C	x	132.6	61.852	65.2122			
C		86.2	108.6585	108.4674			
C		68.3	118.3004	116.3889			
C		19.3	168.2656	171.6506			
C		56.1	133.7827	134.3884			
H	x	6.03	26.6634	**26.6698**			
H	x	7.45	25.0653	**25.0358**			
H	x	7	25.7955	**25.7968**			
H	x	7.49	25.1192	**25.1125**			
H	x	7.63	25.0339	**25.0338**			
H	x	7.27	24.8308	**24.8269**			
H	x	7.3	24.8165	**24.8111**			
H	x	7.62	24.8068	**24.8062**			
H	x	7.09	25.7791	**25.7654**			
H	x	6.91	25.3703	**25.3557**			
H	x	6.56	26.1716	**26.1576**			
H	x	6.47	26.2534	**26.2459**			
H	x	6.34	25.365	**25.3028**			
H	x	5.7	26.5598	**26.499**			
H		3.46	28.2939	**28.3398**			
H		3.62	28.2371	**27.9394**			
H		1.02	30.6237	**31.138**			
H		1.02	31.2164	**31.1664**			
H		1.02	30.8978	**30.9211**			
H		3.21	28.5536	**28.5931**			
H		3.21	29.0011	**29.0169**			
H		3.21	28.5287	**28.5493**			

Figure S35. Experimental chemical shifts and calculated shielding tensors for PD4+ probability analysis for compound **6** (isomer **6a**: rel-(18**R**,19**R**)-**6**; isomer **6b**: rel-(18**S**,19**R**)-**6**).

Functional		Solvent?		Basis Set		Type of Data	
B3LYP		PCM		6-31G(d)		Shielding Tensors	

		DP4+	98.70%	1.30%	–	–	–
Nuclei	sp2?	xperiments	Isomer 1	Isomer 2	Isomer 3	Isomer 4	Isomer 5
C	x	167.6	28.7037	28.6332			
C	x	122.4	74.762	74.9165			
C	x	144.6	50.9766	50.8392			
C	x	128.4	65.0218	65.0994			
C	x	136.9	54.051	53.91			
C	x	133.7	59.3529	59.3276			
C	x	126.3	66.7804	66.7627			
C	x	127.7	68.7908	68.7517			
C	x	129	67.4192	67.4115			
C	x	125.9	67.3327	67.2273			
C	x	135.7	55.3389	55.3365			
C	x	128.9	58.1423	57.8941			
C	x	131.6	66.7367	66.6896			
C	x	133.3	65.0501	64.8014			
C	x	133.3	65.9629	65.9585			
C	x	133.2	69.5645	67.651			
C	x	132	61.852	65.2111			
C		86.1	108.6584	108.4673			
C		68	118.3004	116.393			
C		18.7	168.2656	171.6504			
C		56.2	133.7828	134.3901			
H	x	6.01	26.6633	26.6698			
H	x	7.45	25.0653	25.036			
H	x	7.01	25.7955	25.7967			
H	x	7.49	25.1192	25.1121			
H	x	7.63	25.0339	25.0338			
H	x	7.26	24.8308	24.8269			
H	x	7.31	24.8165	24.8111			
H	x	7.62	24.8068	24.806			
H	x	7.09	25.7791	25.7653			
H	x	6.89	25.3703	25.3551			
H	x	6.52	26.1716	26.1577			
H	x	6.48	26.2534	26.2456			
H	x	6.34	25.365	25.3024			
H	x	5.67	26.5598	26.499			
H		3.47	28.2939	28.3396			
H		3.62	28.2371	27.9394			
H		0.96	30.8978	30.921			
H		0.96	30.6236	31.1379			
H		0.96	31.2164	31.1663			
H		3.21	29.0011	28.5488			
H		3.21	28.5287	28.5931			
H		3.21	28.5537	29.0166			

Figure S36. DP4+ probability analysis of **5** with isomers **5a** and **5b.**

Functional	Solvent?	Basis Set	Type of Data
B3LYP	PCM	6-31G(d)	Shielding Tensors

	Isomer 1	Isomer 2	Isomer 3	Isomer 4	Isomer 5	Isomer 6
sDP4+ (H data)	97.24%	2.76%	–	–	–	–
sDP4+ (C data)	95.32%	4.68%	–	–	–	–
sDP4+ (all data)	99.86%	0.14%	–	–	–	–
uDP4+ (H data)	5.28%	94.72%	–	–	–	–
uDP4+ (C data)	96.44%	3.56%	–	–	–	–
uDP4+ (all data)	60.13%	39.87%	–	–	–	–
DP4+ (H data)	66.29%	33.71%	–	–	–	–
DP4+ (C data)	99.82%	0.18%	–	–	–	–
DP4+ (all data)	99.91%	0.09%	–	–	–	–

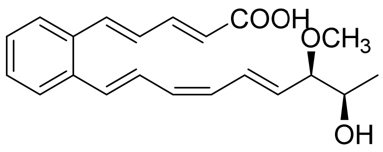

5a

DP4+:99.91%

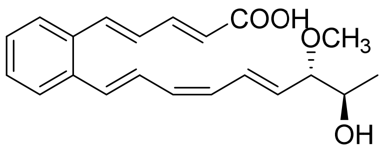

5b

DP4+:0.09%

5a: rel-(18*S*,19*S*), **5b:** rel-(18*S*,19*R*)

Figure S37. DP4+ probability analysis of **6** with isomers **6a** and **6b.**

Functional	Solvent?	Basis Set	Type of Data
B3LYP	PCM	6-31G(d)	Shielding Tensors

	Isomer 1	Isomer 2	Isomer 3	Isomer 4	Isomer 5	Isomer 6
sDP4+ (H data)	95.88%	4.12%	–	–	–	–
sDP4+ (C data)	86.26%	13.74%	–	–	–	–
sDP4+ (all data)	99.32%	0.68%	–	–	–	–
uDP4+ (H data)	9.12%	90.88%	–	–	–	–
uDP4+ (C data)	83.86%	16.14%	–	–	–	–
uDP4+ (all data)	34.28%	65.72%	–	–	–	–
DP4+ (H data)	70.04%	29.96%	–	–	–	–
DP4+ (C data)	97.02%	2.98%	–	–	–	–
DP4+ (all data)	98.70%	1.30%	–	–	–	–

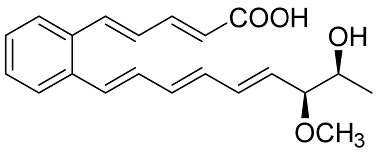

6a

DP4+:98.70%

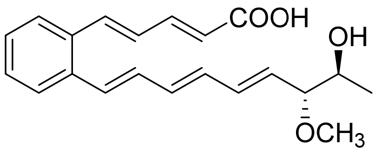

6b

DP4+:1.3%

6a: rel-(18*R*,19*R*). **6b:** rel-(18*S*,19*R*)

Table S1. Experimental (Exp.) and calculated (Cal.)^{1}H and ^{13}C chemical shift values of **5** and its possible isomers **5a** and **5b** used for DP4+ analysis.

Carbon	Exp.	Cal.	Cal.	Proton	Exp.	Cal.	Cal.
	**5**	**5a**	**5b**		**5**	**5a**	**5b**
1	167.6	168.2964	168.368	2	6.03	5.583425	5.589823
2	122.4	122.238	122.0845	3	7.45	7.216935	7.187444
3	144.6	146.0234	146.1583	4	7	6.456163	6.457463
4	128.4	131.9782	131.8957	5	7.49	7.140258	7.13356
5	136.9	142.949	143.0843	7	7.63	7.218934	7.218834
6	133.7	137.6471	137.6657	8	7.27	7.425772	7.421873
7	126.3	130.2196	130.2361	9	7.3	7.441568	7.436169
8	127.7	128.2092	128.2495	10	7.62	7.446466	7.445866
9	128.9	129.5808	129.5904	12	7.09	6.487554	6.473858
10	125.8	129.6673	129.7812	13	6.91	6.897131	6.882535
11	135.7	141.6611	141.6625	14	6.56	6.095471	6.081476
12	128.9	138.8579	139.1068	15	6.47	6.007198	5.9997
13	131.6	130.2634	130.3079	16	6.34	6.950015	6.887834
14	133.4	131.9499	132.2047	17	5.7	5.754174	5.693392
15	133.3	131.0371	131.0416	18	3.46	3.913926	3.959812
16	133.2	127.4355	129.3464	19	3.62	4.314206	4.016595
17	132.6	135.148	131.7878	20	1.02	1.116565	1.630711
18	86.2	88.3415	88.5326	20	1.02	1.088174	1.038189
19	68.3	78.6996	80.6111	20	1.02	1.3334	1.356693
20	19.3	28.7344	25.3494	21-OCH3	3.21	3.660702	3.70019
21-OCH3	56.1	63.2173	62.6116	21-OCH3	3.21	3.237029	3.252824
				21-OCH3	3.21	3.704489	3.725082

Table S2. Experimental (Exp.) and calculated (Cal.)^{1}H and ^{13}C chemical shift values of **6** and its possible isomers **6a** and **6b** used for DP4+ analysis.

Carbon	Exp.	Cal.	Cal.	Proton	Exp.	Cal.	Cal.
	**6**	**6a**	**6b**		**6**	**6a**	**6b**
1	167.6	168.2963	168.3668	2	6.01	5.589823	5.583425
2	122.4	122.238	122.0835	3	7.45	7.187444	7.216735
3	144.6	146.0234	146.1608	4	7.01	6.457463	6.456263
4	128.4	131.9782	131.9006	5	7.49	7.13356	7.140658
5	136.9	142.949	143.09	7	7.63	7.218834	7.218934
6	133.7	137.6471	137.6724	8	7.26	7.421873	7.425772
7	126.3	130.2196	130.2373	9	7.31	7.436169	7.441568
8	127.7	128.2092	128.2483	10	7.62	7.445866	7.446666
9	129	129.5808	129.5885	12	7.09	6.473858	6.487654
10	125.9	129.6673	129.7727	13	6.89	6.882535	6.897731
11	135.7	141.6611	141.6635	14	6.52	6.081476	6.095371
12	128.9	138.8577	139.1059	15	6.48	5.9997	6.007498
13	131.6	130.2633	130.3104	16	6.34	6.887834	6.950415
14	133.3	131.9499	132.1986	17	5.67	5.693392	5.754174
15	133.3	131.0371	131.0415	18	3.47	3.959812	3.914126
16	133.2	127.4355	129.349	19	3.62	4.016595	4.314206
17	132	135.148	131.7889	20	0.96	1.356693	1.3335
18	86.1	88.3416	88.5327	20	0.96	1.630811	1.116665
19	68	78.6996	80.607	20	0.96	1.038189	1.088274
20	18.7	28.7344	25.3496	21-OCH3	3.21	3.252824	3.704989
21-OCH3	56.2	63.2172	62.6099	21-OCH3	3.21	3.725082	3.660702
				21-OCH3	3.21	3.70009	3.237329

Table S3. DFT-optimized structures and thermodynamic parameters for low-energy conformers of **5a** and **5b.**

Conformers	Conf. **5a**	Conf. **5b**
DFT-optimized structures	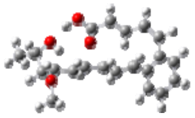	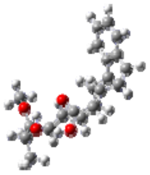
Population	97.7%	94.90%
Total energy (a.u.)	-1115.603516	-1115.604207

Table S4. DFT-optimized structures and thermodynamic parameters for low-energy conformers of **6a** and **6b.**

Conformers	Conf. **6a**	Conf. **6b**
DFT-optimized structures	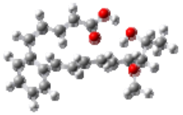	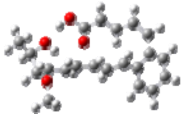
Population	93.10%	94.80%
Total energy (a.u.)	-1115.603516	-1115.604207

Table S5. Optimized Z-matrixes of **5a** in the gas phase (Å) at B3LYP/6-31G(d) level.

		**5a**	
C	5.336832	-1.52326	-1.61525
C	4.412528	-2.4755	-1.1747
C	3.466341	-2.129	-0.21246
C	3.396423	-0.82643	0.317187
C	4.304218	0.153988	-0.17049
C	5.282119	-0.22713	-1.10671
C	4.25911	1.572199	0.249963
C	3.175091	2.385246	0.243674
C	1.860854	2.012385	-0.24458
C	0.729356	2.718339	-0.04284
C	-0.56797	2.187144	-0.51112
O	-1.61136	2.88104	-0.02506
O	-0.70062	1.204563	-1.23474
C	2.449216	-0.52435	1.404948
C	1.154841	-0.91235	1.450642
C	0.26855	-0.58821	2.553459
C	-1.09067	-0.60061	2.53399
C	-1.92946	-0.86433	1.380022
C	-3.27281	-0.76719	1.394495
C	-4.16245	-0.9304	0.185203
C	-4.67306	0.427441	-0.36352
C	-5.75996	0.257554	-1.4252
O	-3.59	1.210464	-0.87768

O	-3.51214	-1.57358	-0.90608
C	-3.41935	-2.9879	-0.80065
H	6.092441	-1.788	-2.34889
H	4.446543	-3.49095	-1.55869
H	2.788871	-2.88439	0.1743
H	5.986478	0.521007	-1.4615
H	5.211019	2.006396	0.55476
H	3.295085	3.402382	0.613626
H	1.772699	1.086547	-0.80557
H	0.71195	3.642061	0.528576
H	-2.43953	2.398846	-0.27886
H	2.830605	0.100344	2.212151
H	0.73793	-1.44323	0.597101
H	0.753945	-0.26971	3.475007
H	-1.61016	-0.32682	3.451399
H	-1.44574	-1.09655	0.434795
H	-3.7862	-0.50441	2.320491
H	-5.05617	-1.51051	0.474982
H	-5.07261	1.001781	0.480446
H	-5.38298	-0.32307	-2.27181
H	-6.63395	-0.25731	-1.00933
H	-6.07689	1.239319	-1.78764
H	-2.955	0.643842	-1.35148
H	-2.85697	-3.29404	0.090324
H	-4.41955	-3.44426	-0.76732
H	-2.89742	-3.33519	-1.69476

Table S6. Optimized Z-matrixes of **5b** in the gas phase (Å) at B3LYP/6-31G(d) level.

		5b	
C	5.458025	-1.42651	-1.39417
C	4.534906	-2.40055	-1.00159
C	3.523412	-2.07332	-0.10088
C	3.38679	-0.76945	0.412343
C	4.294542	0.232304	-0.02984
C	5.33728	-0.12859	-0.902
C	4.184536	1.652561	0.370751
C	3.079689	2.433294	0.291499
C	1.808312	2.014954	-0.26724
C	0.64695	2.690886	-0.14966
C	-0.6021	2.109765	-0.68446
O	-1.69383	2.78656	-0.28832
O	-0.66067	1.102808	-1.38396
C	2.371427	-0.48744	1.44282
C	1.086682	-0.90805	1.417822
C	0.134552	-0.6106	2.472382
C	-1.22102	-0.66313	2.382652
C	-1.99174	-0.94041	1.185332
C	-3.33823	-0.90908	1.140205
C	-4.15986	-1.09623	-0.11259
C	-4.6586	0.225508	-0.75094
C	-5.50501	1.0845	0.17793
O	-3.56596	1.025537	-1.21023

O	-3.44386	-1.75209	-1.15769
C	-3.30749	-3.15828	-1.00253
H	6.263518	-1.67574	-2.07852
H	4.619301	-3.41739	-1.37396
H	2.84485	-2.84438	0.251493
H	6.040843	0.636169	-1.22116
H	5.104608	2.117794	0.723667
H	3.148694	3.45793	0.653633
H	1.780055	1.077177	-0.81423
H	0.567392	3.625031	0.399046
H	-2.4896	2.272351	-0.57846
H	2.691839	0.147519	2.268373
H	0.73045	-1.45015	0.544095
H	0.561743	-0.28441	3.41972
H	-1.79467	-0.41511	3.274863
H	-1.4531	-1.12966	0.260463
H	-3.90024	-0.69961	2.050282
H	-5.06381	-1.68133	0.132917
H	-5.25848	-0.07403	-1.62268
H	-6.3768	0.526214	0.535528
H	-4.92482	1.423263	1.041486
H	-5.86268	1.966629	-0.36063
H	-2.88899	0.466964	-1.63187
H	-2.75417	-3.5168	-1.873
H	-4.29299	-3.64633	-0.9768
H	-2.75665	-3.41738	-0.08979

Table S7. Optimized Z-matrixes of **6a** in the gas phase (Å) at B3LYP/6-31G(d) level.

		**6a**	
C	-5.33683	-1.52326	-1.61525
C	-4.41253	-2.4755	-1.1747
C	-3.46634	-2.129	-0.21246
C	-3.39642	-0.82643	0.317187
C	-4.30422	0.153988	-0.17049
C	-5.28212	-0.22713	-1.10671
C	-4.25911	1.572199	0.249963
C	-3.17509	2.385246	0.243673
C	-1.86085	2.012385	-0.24458
C	-0.72936	2.718338	-0.04284
C	0.567966	2.187144	-0.51112
O	1.611356	2.88104	-0.02506
O	0.700621	1.204563	-1.23474
C	-2.44922	-0.52435	1.404948
C	-1.15484	-0.91235	1.450642
C	-0.26855	-0.58821	2.553459
C	1.090667	-0.60061	2.53399
C	1.92946	-0.86433	1.380022
C	3.272814	-0.76719	1.394495
C	4.162453	-0.9304	0.185203
C	4.67306	0.427441	-0.36352
C	5.759963	0.257554	-1.4252
O	3.589995	1.210464	-0.87768

O	3.512137	-1.57358	-0.90609
C	3.41935	-2.9879	-0.80065
H	-6.09244	-1.788	-2.34889
H	-4.44654	-3.49095	-1.55869
H	-2.78887	-2.88439	0.1743
H	-5.98648	0.521007	-1.4615
H	-5.21102	2.006396	0.554759
H	-3.29509	3.402382	0.613626
H	-1.7727	1.086547	-0.80557
H	-0.71195	3.642061	0.528577
H	2.439529	2.398846	-0.27886
H	-2.83061	0.100344	2.212151
H	-0.73793	-1.44323	0.597101
H	-0.75395	-0.26971	3.475007
H	1.610158	-0.32682	3.451399
H	1.445741	-1.09655	0.434795
H	3.786198	-0.50441	2.320491
H	5.056168	-1.51051	0.474982
H	5.072613	1.001781	0.480446
H	6.076888	1.239319	-1.78764
H	5.382976	-0.32307	-2.27181
H	6.633953	-0.25731	-1.00933
H	2.954996	0.643842	-1.35148
H	4.419554	-3.44426	-0.76733
H	2.897422	-3.33519	-1.69476
H	2.856971	-3.29404	0.090323

Table S8. Optimized Z-matrixes of **6b** in the gas phase (Å) at B3LYP/6-31G(d) level.

		**6b**	
C	5.457698	-1.42594	-1.39469
C	4.534629	-2.40012	-1.00236
C	3.523227	-2.07322	-0.1014
C	3.386638	-0.76955	0.412228
C	4.294287	0.232404	-0.02974
C	5.336973	-0.12817	-0.90212
C	4.184274	1.652577	0.371283
C	3.079452	2.433376	0.292082
C	1.808094	2.015084	-0.26684
C	0.646696	2.690989	-0.14939
C	-0.6023	2.1099	-0.68434
O	-1.69406	2.786849	-0.2884
O	-0.66085	1.102918	-1.38378
C	2.37138	-0.48772	1.442948
C	1.086712	-0.90861	1.4181
C	0.134533	-0.61137	2.472726
C	-1.22102	-0.6637	2.382871
C	-1.99168	-0.94088	1.185485
C	-3.33816	-0.90911	1.140238
C	-4.15957	-1.09624	-0.11272
C	-4.65842	0.225388	-0.75115
C	-5.50498	1.084389	0.177601
O	-3.56579	1.025458	-1.21039
O	-3.44309	-1.75185	-1.15768

C	-3.30644	-3.15801	-1.00268
H	6.263152	-1.67499	-2.07916
H	4.619046	-3.41683	-1.37508
H	2.844615	-2.84432	0.250783
H	6.040508	0.636723	-1.22103
H	5.104337	2.11772	0.724351
H	3.148349	3.457933	0.654414
H	1.779956	1.07734	-0.81387
H	0.567081	3.625117	0.399335
H	-2.48986	2.272645	-0.57847
H	2.691792	0.147268	2.268469
H	0.73045	-1.45059	0.544301
H	0.561739	-0.28541	3.420146
H	-1.79474	-0.4156	3.275023
H	-1.45304	-1.13049	0.260691
H	-3.90023	-0.69924	2.050184
H	-5.0634	-1.68162	0.132595
H	-5.25825	-0.07428	-1.6229
H	-5.86285	1.966307	-0.36113
H	-6.37664	0.525958	0.535297
H	-4.92483	1.423319	1.041125
H	-2.88867	0.466832	-1.6317
H	-2.75582	-3.41712	-0.08979
H	-2.75272	-3.51625	-1.873
H	-4.29183	-3.64634	-0.97732

Table S9. Cytotoxicity of **1–9** against RAW264.7 and MCF-7 cells (IC_{50} μM).

Cell lines	RAW264.7	MCF-7
1	>200	>20
2	>200	>20
3	>200	>20
4	>200	>20
5	>200	>20
6	>200	>20
7	>200	>20
8	>200	>20
9	>200	>20
ADR(μM)	1.492±0.09	0.749±0.13
